# Proteomic Analysis Reveals the Composition of Glutamatergic Organelles of Auditory Inner Hair Cells

**DOI:** 10.1016/j.mcpro.2023.100704

**Published:** 2023-12-20

**Authors:** Andreia P. Cepeda, Momchil Ninov, Jakob Neef, Iwan Parfentev, Kathrin Kusch, Ellen Reisinger, Reinhard Jahn, Tobias Moser, Henning Urlaub

**Affiliations:** 1 Bioanalytical Mass Spectrometry Group, Max Planck Institute for Multidisciplinary Sciences, Göttingen, Germany; 2 Department of Clinical Chemistry, University Medical Center Göttingen, Göttingen, Germany; 3 Institute for Auditory Neuroscience and InnerEarLab, University Medical Center Göttingen, Göttingen, Germany; 4 Auditory Neuroscience & Synaptic Nanophysiology Group Max-Planck-Institute for Multidisciplinary Sciences, Göttingen, Germany; 5 Functional Auditory Genomics Group, Institute for Auditory Neuroscience and InnerEarLab, University Medical Center Göttingen, Göttingen, Germany; 6 Gene Therapy for Hearing Impairment and Deafness, Department for Otolaryngology, Head & Neck Surgery, University Hospital Tübingen, Tübingen, Germany; 7 Laboratory of Neurobiology, Max Planck Institute for Multidisciplinary Sciences, Göttingen, Germany; 8 Cluster of Excellence "Multiscale Bioimaging: from Molecular Machines to Networks of Excitable Cells" (MBExC), University of Göttingen, Göttingen, Germany

**Keywords:** vesicles, inner hair cells, trafficking, VGluT3, proteomics

## Abstract

In the ear, inner hair cells (IHCs) employ sophisticated glutamatergic ribbon synapses with afferent neurons to transmit auditory information to the brain. The presynaptic machinery responsible for neurotransmitter release in IHC synapses includes proteins such as the multi-C_2_-domain protein otoferlin and the vesicular glutamate transporter 3 (VGluT3). Yet, much of this likely unique molecular machinery remains to be deciphered. The scarcity of material has so far hampered biochemical studies which require large amounts of purified samples. We developed a subcellular fractionation workflow combined with immunoisolation of VGluT3-containing membrane vesicles, allowing for the enrichment of glutamatergic organelles that are likely dominated by synaptic vesicles (SVs) of IHCs. We have characterized their protein composition in mice before and after hearing onset using mass spectrometry and confocal imaging and provide a fully annotated proteome with hitherto unidentified proteins. Despite the prevalence of IHC marker proteins across IHC maturation, the profiles of trafficking proteins differed markedly before and after hearing onset. Among the proteins enriched after hearing onset were VAMP-7, syntaxin-7, syntaxin-8, syntaxin-12/13, SCAMP1, V-ATPase, SV2, and PKCα. Our study provides an inventory of the machinery associated with synaptic vesicle-mediated trafficking and presynaptic activity at IHC ribbon synapses and serves as a foundation for future functional studies.

## Highlights

• Identification of the proteome of trafficking organelles of inner hair cells (IHCs).
• Proteomic profiling reveals age-dependent molecular machinery of organelles.
• Age-dependent changes are consistent with synapse maturation.
• Functional annotation of mature proteome reveals a mixed SV-endosomal signature.

## Graphical Abstract

**Figure:**
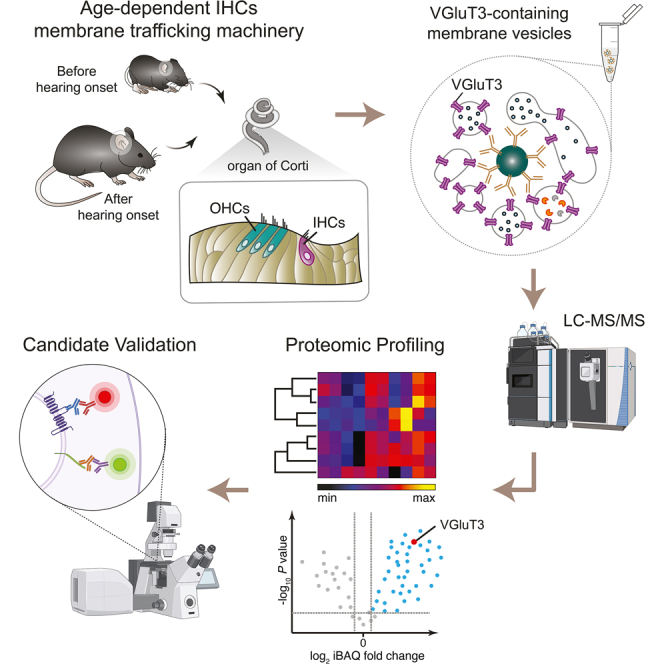

## In Brief

Deciphering ribbon synapses of IHCs has been driven by analysis of human deafness mutations and comparison to well-characterized conventional synapses. Due to the lack of *in vitro* model systems, most studies have targeted specific proteins in experiments with mutant mice. Yet the mapping of protein networks in IHCs has remained challenging due to limited material for biochemical and omics studies. Our study represents the first attempt to obtain a complete map of the trafficking proteome of IHCs under native conditions.

Sound encoding occurs with remarkable precision, reliability, and dynamics over prolonged periods of stimulation (1, 2, 3, 4, 5, 6, 7, 8, 9). Sensory inner hair cells (IHCs) of the inner ear carry out an essential role in this process, transforming sound-evoked mechanical vibrations into a neural code *via* synaptic transmission to spiral ganglion neurons (SGNs). Impairment or loss of synaptic sound encoding, *i.e.*, synaptic hearing impairment or auditory synaptopathy, results from genetic defects, exposure to loud noise, and ototoxic drugs as well as from aging (8, 10, 11).

Afferent IHC synapses differ from conventional synapses, including the efferent synapses of the cochlea, at both a structural and molecular level, suggesting an unconventional mechanism of neurotransmitter release to serve the specific needs of sound encoding. Each presynaptic active zone (AZ) of IHCs features a large, electron-dense structure called “synaptic ribbon” that is primarily constituted by the protein RIBEYE and tethers a halo of synaptic vesicles (SVs) (4, 12, 13, 14, 15, 16). Functions attributed to IHC synaptic ribbons include establishing a large number of vesicular release sites and enabling high rates of vesicle replenishment (15, 16, 17, 18, 19, 20, 21). Most studies, performed in rodents, report that upon maturation of IHC AZs smaller and often multiple ribbons give way to one or two larger ribbons (22, 23, 24, 25, 26). This morphological AZ maturation is accompanied by a switch from a “spontaneous” pre-sensory activity to acoustically evoked (sensory) activity in the SGNs. The landmark of this functional maturation is hearing onset, occurring at postnatal day (P) 12 in mice (27).

Most canonical synaptic proteins crucial for Ca^2+^-triggered exocytosis at synapses of the central nervous system (CNS), *e.g.*, SNAREs, synaptotagmins, Munc13, and complexins, are either absent or largely dispensable for exocytosis in mature IHCs but are expressed in the conventional efferent synapses formed onto the postsynaptic neurites of SGNs underneath the IHCs (4, 28, 29, 30, 31, 32, 33). Instead, IHCs express the multi-C_2_ domain protein otoferlin, and defects of the coding *Otof* gene lead to auditory synaptopathy (32, 34, 35, 36, 37, 38, 39). Aside from its likely role as Ca^2+^ sensor for exocytosis in IHCs (36, 40, 41), otoferlin also promotes SV replenishment and contributes to coupling exocytosis to endocytosis (39, 42, 43, 44). Another hallmark of IHC synapses is the presence of the unconventional vesicular glutamate transporter 3 (VGluT3), also known as solute carrier family 17 member 8 (Slc17a8), with its expression in the cochlea being largely restricted to IHCs (45). VGluT3 was initially discovered in the brains of rodents and humans, it presents ∼70% sequence homology with VGluT1 and VGluT2 (expressed in all glutamatergic neuronal populations). Glutamate transport by VGluT3 seems to be mostly similar to that of the canonical glutamate transporters (46, 47, 48, 49, 50). Its localization in the brain, however, seems to be very restricted and exclusively linked to a subset of cholinergic and serotonergic neurons (49, 51), predicting a different role for glutamate signaling in VGluT3-positive neurons. VGluT3 knockout mice are profoundly deaf due to the absence of glutamate release from IHCs. Early loss of SGNs (52, 53) predicted an additional developmental role for glutamate release, required for SGN survival (52, 53) and for maintaining molecular SGN subtype specification (54).

While a few proteins governing synaptic transmission and vesicle trafficking events in IHCs are known (review in (9, 36, 43, 55, 56, 57, 58, 59, 60, 61)), attaining a comprehensive account of the protein networks participating in these events remained difficult, mostly due to the effort required in harvesting sufficient cochlear material for analysis. Transcriptomic and proteomic analyses of isolated inner ear hair cell populations (62, 63, 64, 65, 66, 67) have provided an overall expression profile of an entire cell but lacked detailed subcellular localization information. Subcellular fractionation procedures rely on differential and density-gradient centrifugations and can be complemented with affinity-based approaches. Protocols for the purification of SVs from CNS synapses have been successfully developed (68, 69, 70) and further improved (71, 72, 73, 74). Although laborious, they result in material of considerably high purity and have also been used to isolate SV subsets and endosomes. While such procedures were developed for the brain already in the 1960s, and have since been frequently used, the same does not hold true for the cochlea. Efforts have been made in this direction, with attempts to isolate *e.g.* ribbon material (14, 75) and otoferlin-associated proteins (43). These studies were good initial efforts at subcellular isolation, albeit the use of detergents during fractionation defeats the main goal of obtaining virtually intact unsolubilized subcellular components for downstream analysis.

**Fig. 1:**
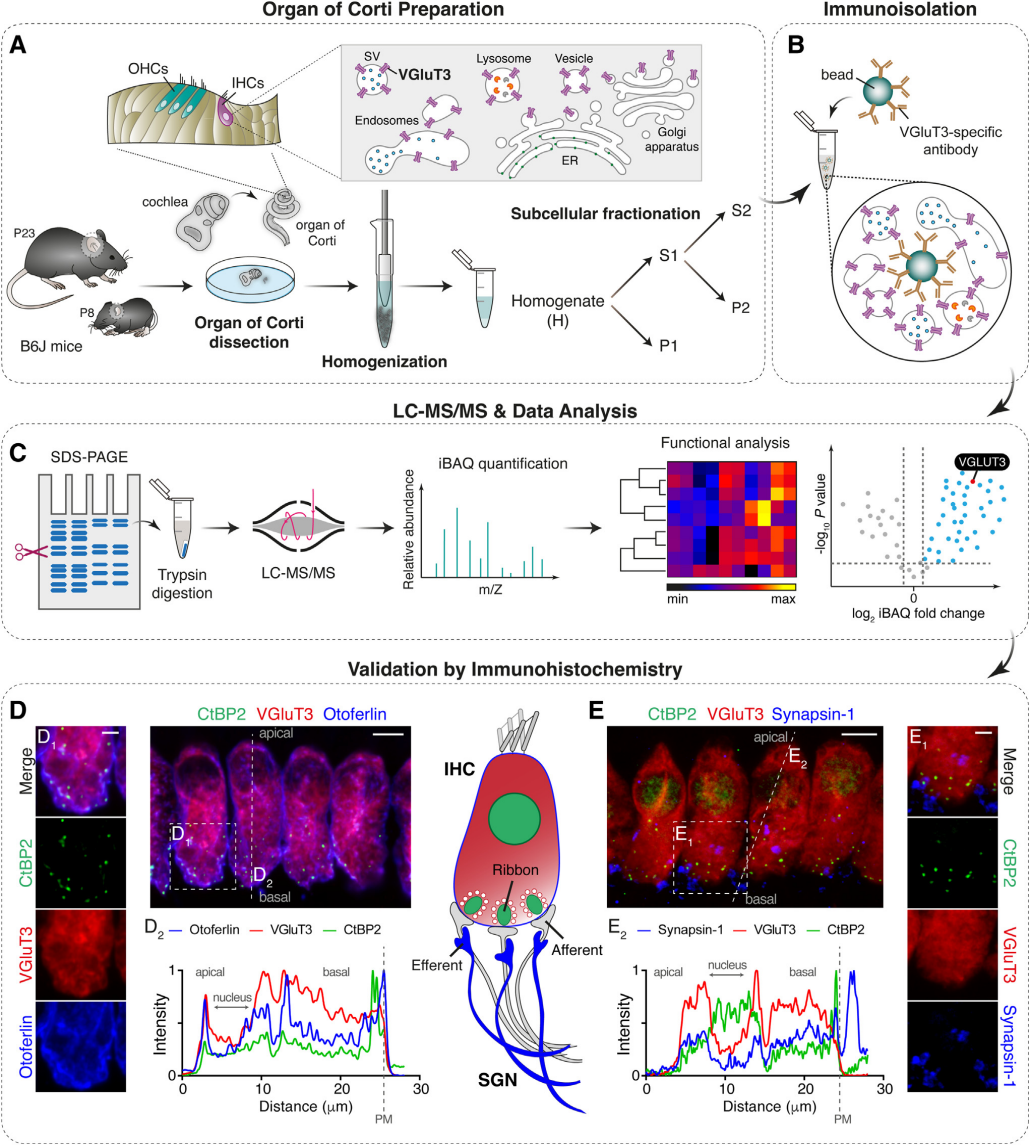
**Illustration of the workflow used for the identification of the VGluT3-associated mouse IHC proteome.***A*, organs of Corti of immature (P8, before hearing onset) and mature (P23, after hearing onset) wild-type mice were explanted, homogenized, and fractionated by differential centrifugation. The organ of Corti is the auditory sensory epithelium and contains one row of IHCs and three rows of OHCs. VGluT3 is primarily expressed in trafficking organelles (vesicles and endolysosomal compartments) of IHCs. *B*, IHC VGluT3-positive organelles were immunoisolated using a VGluT3-specific antibody. *C*, subcellular fractions and immunoisolates were processed in a conventional bottom-up proteomics workflow. *D* and *E*, positive protein hits from the LC-MS/MS analyses were validated by immunohistochemistry using antibodies against VGluT3 and otoferlin to label IHCs, an antibody against RIBEYE/CtBP2 to label synaptic ribbons, and sometimes an antibody against synapsin-1 to label efferent synapses onto SGNs. *D*, representative mature IHCs at P15 immunolabeled for CtBP2 (*green*), VGluT3 (*red*), and otoferlin (*blue*) (scale bar: 5 μm); *D1* shows higher magnification views of the basal region of one IHC (scale bar: 2 μm). The profile plot shows otoferlin’s immunofluorescence signal at the PM (*D2*). *E*, representative P15 IHCs immunolabeled for CtBP2 (*green*), VGluT3 (*red*), and synapsin-1 (*blue*) (scale bar: 5 μm); *E*_*1*_ shows higher magnification views of the basal region of one IHC (scale bar: 2 μm); the profile plot shows synapsin-1 signal beyond the IHC basolateral PM (*E2*). In (*D* and *E*), maximum intensity projections of 5 to 10 confocal optical sections. In (*D*_*2*_ and *E*_*2*_), fluorescence intensity line profiles through the longitudinal axis in the mid-region of a representative IHC, from apex to base (5–10 optical sections). ER, endoplasmic reticulum; IHC, inner hair cell; LC-MS/MS, mass spectrometry; OHC, outer hair cell; PM, plasma membrane; SV, synaptic vesicle; SGN, spiral ganglion neuron; VGluT3, vesicular glutamate transporter three.

Building on established subcellular fractionation protocols and taking advantage of the unique expression pattern of VGluT3 in the mammalian cochlea, we have developed a workflow for isolating IHC SVs and other VGluT3-positive vesicular organelles (Fig. 1). To achieve a detailed and reliable characterization, we used a proteomic approach that combines subcellular fractionation and antibody-based affinity purification together with label-free quantitation (LFQ) liquid chromatography–mass spectrometry (LC-MS). To validate protein expression in IHCs, selected mass spectrometry hits were characterized by immunohistochemistry and functional analysis. Comparison between before and after hearing onset allowed us to define developmental changes within the VGluT3-vesicular IHC proteome and led to the identification of proteins potentially important to presynaptic IHC function after synapse maturation. This comprehensive proteome provides a roadmap for future ultrastructural and functional studies of candidate proteins.

## Experimental Procedures

### Experimental Design and Statistical Rationale

We first set out to establish a protocol for the subcellular fractionation of the mouse organ of Corti and to obtain an IHC-rich fraction. Contrary to previous studies, instead of using intact cochlea we dissected the organ of Corti after isolating the cochlea, improving sample purity by removing most of the cochlear bone and other tissues which could interfere with downstream analysis. Given the scarcity of cochlea-derived material, we used only pre-hearing mice (at P8) for the optimization and validation of this protocol, since dissection of the organ of Corti at this developmental stage is an easier process due to incomplete calcification of the cochlea (76). The protein content of all subcellular fractions was assessed by LC-MS. Due to a very much limited amount of sample material obtained from a large number of mice (see below), the MS data derived from these experiments was obtained from one biological replicate (*i.e.*, one fractionation procedure prepared from ∼150 organs of Corti) measured in duplicate (two technical replicates per subfraction).

Once an IHC-rich subfraction was obtained (S2 fraction), we devised an immunoisolation methodology for the enrichment of IHC trafficking organelles from this fraction. Using the highly IHC-specific expression of VGluT3 in the organ of Corti, and by immobilizing an anti-VGluT3 antibody to magnetic beads, we were able to enrich for IHC vesicular (likely dominated by SVs) and endosomal-like structures and to characterize their protein composition by mass spectrometry. To determine which proteins are specifically enriched in the immunoisolated vesicles, we used as reference (i) the S2 fraction used as starting material for the immunoisolation, and (ii) beads containing immobilized unspecific IgGs that were processed in parallel with the beads containing immobilized VGluT3-antibodies. Mice before and after hearing onset (at P8 and P23, respectively) were analyzed to assess developmental changes. Statistical analyses of MS data were performed on two biological replicates (*i.e.*, two independent fractionation procedures and immunoisolation sets prepared from ∼100 organs of Corti each) measured in duplicate (two technical replicates).

The MaxQuant software was used for intensity-based absolute quantification (iBAQ) of MS data, and bioinformatic and statistical analysis was performed using the Perseus software using the output from MaxQuant searches. Detailed statistical and bioinformatic analyses of proteomics data are described in detail in the “Proteomics Data Analysis” and “Downstream Bioinformatics Analysis of Proteomic Data” sections.

We validated key proteins identified by MS *via* immunohistochemistry on whole-mount explants of organs of Corti and evaluated their presence in IHCs by confocal microscopy. Most of the analysis was performed on P15–25 mice when synapses are matured and vesicular trafficking processes are already well established, and we focused on validating candidate proteins potentially involved in these events.

### Animals

Wild-type C57BL/6J (B6) and mutant (described below) mice of either sex were used. The mice were housed in social groups in individually ventilated cage (IVC) racks in a pathogen-free facility with free access to food and water and 12 h/12 h light/dark cycles. Animal breeding was done in compliance with the German national animal care guidelines as set out in the Animal Welfare Law of the Federal Republic of Germany and was approved by the local animal welfare committee of the University Medical Center Göttingen and the Max Planck Institute for Multidisciplinary Sciences, as well as the animal welfare office of the state of Lower Saxony (LAVES, AZ 19/3134). For conditional deletion of Munc18-1 in IHCs, Munc18-1 conditional knockout mice (Munc18-1^fl/fl^) carrying loxP sites on either side of exon two of Munc18-1 (77) were cross-bred to Math1-creER mice expressing a fusion protein between Cre and the altered ligand-binding domain of the estrogen receptor (CreER) under the control of the Math1 enhancer, leading to inner and outer hair cell-specific expression of creER in the cochlea (78). Cre was activated by intraperitoneal injection of 3 to 4 mg tamoxifen (Sigma T5648) dissolved in corn oil (Sigma C8267) at P0 and P1.

### Subcellular Fractionation of Mouse Organs of Corti

Organs of Corti (OCs) from B6 mice at P8 or P23, *i.e.*, before and after hearing onset, were acutely dissected in ice-cold PBS, pooled, and homogenized in ice-cold sucrose buffer (320 mM sucrose, 4 mM HEPES, pH 7.4, supplemented with protease inhibitors (1 μg/ml PMSF in DMSO; 0.2 mM Pepstatin A in 100% ethanol) using a glass-Teflon homogenizer, with 10 strokes at 900 rpm (adapted from (73, 74, 79)). The resulting homogenate (H) was centrifuged at 1000*g* for 10 min to remove bone, cell debris, and pellet nuclei (P1). The supernatant (S1) was centrifuged for 15 min at 1000*g* to obtain a crude membrane fraction (P2) and a crude vesicular fraction (S2). Vesicular structures were stabilized by supplementation with phosphate-buffered saline (PBS).

### Immunoisolation of VGluT3-Containing Vesicles

A mouse monoclonal antibody directed against VGluT3 (135211, Synaptic Systems) was bound to Protein G magnetic beads (10004D, Thermo Fisher Scientific) following the instructions of the manufacturer. Briefly, beads were saturated with antibody for 1 h with gentle end-over-end mixing, and excess antibody was washed out by washing three times for 10 min with PBS. Antibody-coated beads were then added to the vesicle fraction (S2) in the presence of protease inhibitors (1 μg/ml PMSF in DMSO; 0.2 mM Pepstatin A in 100% ethanol). Magnetic beads were separated from the immunodepleted supernatant and washed three times with PBS. Bound vesicles were eluted in 4× NuPAGE LDS Sample Buffer supplemented with 10% beta-mercaptoethanol.

### Sample Preparation and In-Gel Digestion for MS-Based Proteomics

Fractionationated and immunoisolatated samples were resuspended in 4× NuPAGE LDS Sample Buffer supplemented with 10% beta-mercaptoethanol and loaded onto NuPAGE 4–12% Bis-Tris Gels (Thermo Fisher Scientific). Following detection by InstantBlue Coomassie Protein Stain (Expedeon), all lanes were excised, cut into approximately 1 mm^2^ pieces, and subjected to in-gel reduction with dithiothreitol, alkylation with iodoacetamide, and overnight trypsinization (80). Tryptic peptides were extracted, dried, and reconstituted in 4% (v/v) acetonitrile, 0.05% (v/v) trifluoroacetic acid.

### LC-MS/MS Analysis

Each gel-derived fraction was analyzed as technical duplicates. Individual fractions were analyzed by LC-MS/MS in an Orbitrap Exploris 480 mass spectrometer (Thermo Fisher Scientific) coupled to a Dionex UltiMate 3000 UHPLC system (Thermo Fisher Scientific) equipped with an in-house-packed C18 column (ReproSil-Pur 120 C18-AQ, 1.9 μm pore size, 75 μm inner diameter, 30 cm length; Dr Maisch GmbH).

Peptides obtained after digestion of proteins in each subcellular fraction (H, P1, S1, P2, S2) were separated using a 118-min gradient (start at 5% B for 3 min, increase to 8% B in 7 min, followed by 8–35% B for 90 min, 35–55% B for 7 min, 90% B for 4 min, reequilibration to 5% B for 6 min) at a flow rate of 300 nl/min, using 0.1% (v/v) formic acid in water as mobile phase A and 80% (v/v) acetonitrile, 0.08% (v/v) formic acid in water as mobile phase B. Eluting peptides were analyzed in positive mode by a data-dependent acquisition method (data-dependent mode: cycle time; time between master scans: 3 s). MS1 spectra were acquired with a resolution of 120,000 FWHM (full width at half maximum) covering a mass range of 350 to 1600 *m/z*, 300% automatic gain control (AGC) target (100% = 1 × 10^6^), and 25 ms maximum injection time. The intensity threshold was set to 5 × 10^3^. Precursor ions were isolated using a 1.4 *m/z* isolation window, and a higher-energy collisional dissociation (HCD) of 28% was applied to obtain fragmented spectra. Only precursors with charge states from 2+ to 6+ were considered, and the dynamic exclusion was set to 25 s. MS2 spectra were acquired with a resolution of 15,000 FWHM, 75% AGC target (100% = 1 × 10^5^), 32 ms maximum injection time, and a fixed first mass of 110 *m/z*.

For MS analysis of enriched proteins after final VGluT3 immunoisolation, immunoisolation using control IgG, and input S2 used in immunoisolation experiments, peptides derived from these samples after digestion were separated using a 58-min gradient (start at 5% B for 0.3 min, increase to 10% B in 2.7 min, followed by 10–36% B for 37 min, 36–45% B for 5.9 min, 45–90% B for 2.1 min, 90% B for 4 min, reequilibration to 5% B for 5 min) at a flow rate of 300 nl/min, using 0.1% (v/v) formic acid in water as mobile phase A and 80% (v/v) acetonitrile, 0.08% (v/v) formic acid in water as mobile phase B. Eluted peptides were analyzed in positive mode using a data-dependent acquisition method (selecting the top 30 most abundant precursors for higher energy collision dissociation). MS1 spectra were acquired at a resolution of 60,000 FWHM covering a mass range of 350 to 1600 *m/z*, 100% AGC target (1 × 10^6^), and 40 ms maximum injection time. The intensity threshold was set to 1 × 10^4^. Precursor ions were isolated using a 1.6 *m/z* isolation window, and an HCD of 30% was applied for fragmentation. Only precursors with charge states from 2+ to 6+ were considered, and the dynamic exclusion was set to 20 s. MS2 spectra were acquired with a resolution of 15,000 FWHM, 100% AGC target (1 × 10^5^), 54 ms maximum injection time, and a fixed first mass of 120 *m/z*.

### Proteomics Data Analysis

Raw acquisition files were processed using the MaxQuant computational platform (version 1.6.10.43) (81, 82) with the built-in Andromeda peptide search engine (83). The spectra were searched against the complete *Mus musculus* proteome sequence database generated from UniProt combining all Swiss-Prot and TrEMBL entries (UP000000589, accessed 10 May 2020, 55,398 entries). Trypsin/P was set as a digestion enzyme allowing up to four miscleavages per peptide. Carbamidomethylated cysteines were set as fixed modifications with oxidation of methionines and N-terminal acetylation as variables, and a maximum number of five modifications per site was allowed. False discovery rate (FDR) was kept at 1% at both peptide and protein levels. Peptides with a minimum length of seven amino acids were defined as required for protein identification. The “Match between runs” option with default parameters was enabled, to allow identifications to be transferred to unsequenced MS features in other LC-MS runs. For intensity-based absolute quantification (iBAQ) (84), which normalizes each protein intensity by the corresponding number of identified peptides for any given protein, the “iBAQ” option was enabled.

### Downstream Bioinformatics Analysis of Proteomics Data

Bioinformatic analysis was performed in the Perseus software environment (version 1.6.10.43) (85) using iBAQ values obtained through MaxQuant. Usually, irrelevant protein group identifications were filtered out, that is, identifications only by site or by reverse sequence, and potential contaminants. iBAQ intensities were logarithmized (log_2_).

For organ of Corti fractionation samples (H, P1, S1, P2, S2), the log_2_ iBAQ matrix was reduced based on valid values with a minimum of 100% (2 out of 2) valid values in at least one group. The remaining missing values were imputed based on a normal distribution (downshift of 2.0 and distribution width of 0.5). Multiscatter plots displaying absolute Pearson’s correlation coefficients were generated for quality control purposes. Principal component analysis (PCA) was performed to assess variation between groups. Unsupervised hierarchical clustering analysis (row clustering with Euclidean distance, average linkage, preprocessed with k-means, with 300 clusters and 10 iterations) was performed after z-score normalization. Results were represented in the form of a heatmap of z-scored log_2_-transformed iBAQ intensities of the differentially expressed proteins (rows) across fractions (columns); from yellow (indicating higher abundance) to black (lower abundance), and grey indicating no identifications. The top categorical annotations, supplied as gene ontology (GO) cellular component (CC) and biological process (BP) terms, were assessed with the ShinyGO tool (version 0.75) (86) with the following parameters: species “Mouse”; Fisher’s exact test with FDR correction (*p* < 0.05); top 50 most significant pathways; remove redundant pathways; pathways sorted by enrichment FDR. When ‘Remove redundant pathway’ is selected, similar pathways sharing 95% of genes are represented by the most significant pathway. Due to sample shortage, statistical analyses were performed on two technical replicates per fraction originating from one fractionation procedure (prepared from at least 100 organs of Corti). Final matrices were exported from Perseus and further processed in Microsoft Excel 365.

For immunoisolation-related samples (VGluT3, control IgG, input S2), the log_2_ iBAQ matrix was reduced based on valid values with a minimum of 75% (3 out of 4) valid values in at least one group. The remaining missing values were imputed based on a normal distribution (downshift of 1.4 and distribution width of 0.5). The log_2_ iBAQ matrix was normalized by subtracting the mean log_2_ iBAQ value for a technical replicate from each individual value in that same technical replicate. A two-sample two-sided *t* test (5% FDR, S_0_ = 0) was performed between VGluT3 and control groups using the reduced log_2_ iBAQ matrix after normalization. A volcano plot representation was used to identify the most significant proteins by plotting –log_10_ adjusted *p* value *versus* log_2_ iBAQ fold change between VGluT3 and control groups individually. For 2D comparisons (*i.e.*, to compare protein enrichment in VGluT3 immunoisolates with both control groups), the values in the previous iBAQ matrix were averaged for each group, and the averaged log_2_ iBAQ fold change (VGluT3/Control IgG) *versus* log_2_ iBAQ fold change (VGluT3/Input) was plotted. Proteins with log_2_ iBAQ fold differences >0 were defined to be enriched in VGluT3 immunoisolates over both control IgG and input. To increase confidence in identifications, a cutoff of log_2_ fold difference of 0.7 was set to include only proteins with at least 1.5-fold enrichment. Statistical analyses were performed on two biological replicates with two technical replicates per condition (at least 100 organs of Corti per condition). Final matrices were exported from Perseus and further processed in Microsoft Excel 365. Proteins significantly enriched in VGluT3 immunoisolates over controls (control IgG and input) were ranked by their relative enrichment for both age groups. Relative enrichment was defined as the average of log_2_ iBAQ (VGluT3/Input) and log_2_ iBAQ (VGluT3/Control IgG).

The top 20 GO cellular component terms for proteins enriched in VGluT3 immunoisolates over controls were assessed using the ShinyGO tool (version 0.75) (86) with the following parameters: species “Mouse”; Fisher’s exact test with FDR correction (*p* < 0.05); top 20 most significant pathways; remove redundant pathways; pathways sorted by fold enrichment. Fold enrichment is defined as the percentage of genes in the list belonging to a pathway divided by the corresponding percentage in the “Mouse” background, indicating how drastically genes of a certain pathway are overrepresented). When “Remove redundant pathway” is selected, similar pathways sharing 95% of genes are represented by the most significant pathway.

Enriched proteins in VGluT3 immunoisolates over controls were mapped against the synapse-specific SynGO database (87). Gene names were used as input and the brain was set as background reference. Input data (gene symbols) were first converted to Mouse Genome Informatics identifiers (MGI IDs) using the built-in SynGO ID converter tool and were then mapped to IDs supported by SynGO. SynGO ontology terms that were populated with at least one gene annotation in SynGO were visualized as sunburst plots, an alternative to tree structures, for cellular components (CCs) and were displayed by fold enrichment (–log_10_ Q-value) per term. Top-level terms (*e.g.*, presynapse, postsynapse) are represented by large arbitrarily color-coded internal circles where surface area correlates with the number of proteins in the corresponding category. Functionally annotated subclasses are mapped as second- and higher-level terms in outer circles.

Comprehensive annotation of enriched proteins was performed by searching and manually annotating each protein’s function and cellular localization against relevant databases and literature (UniProt, SynGO, PubMed). All exploratory data analysis was performed in R 4.1.2 (88) using the package “tidyverse” (89), and charts were prepared for display using the packages “ggplot2” (90), “cowplot” (91), and “ggrepel” (92). All data was assembled for display in Adobe Illustrator (Adobe Systems).

### Western Blotting

Subcellular fractions (H, P1, S1, P2, S2), immunodepleted supernatants, and eluted VGluT3-and control immunoisolates were separated by SDS-PAGE on NuPAGE 4–12% Bis-Tris Gels (Thermo Fisher Scientific) and transferred onto nitrocellulose membranes (GE Healthcare Life Sciences). Membranes were probed with primary antibody mouse anti-VGluT3 (135211, Synaptic Systems) and secondary antibodies goat anti-mouse IgG-HRP (115-035-146, Jackson ImmunoResearch) or goat anti-mouse IgG-HRP (115-005-174, Jackson ImmunoResearch). Pierce ECL Plus Western Blotting Substrate (32132, Thermo Fisher Scientific) was used for detection.

### Immunohistochemistry and Confocal Microscopy

The apical turn of organs of Corti P15–25 mice was freshly dissected in PBS, and directly fixed with 4% formaldehyde (FA) in PBS for 45 min at 4 °C.

Immunostainings were performed as previously described (42). The following primary antibodies were used: rabbit anti-VAMP-7 (232003, Synaptic Systems), rabbit anti-syntaxin-6 (110062, Synaptic Systems), rabbit anti-syntaxin-7 (110072, Synaptic Systems), rabbit anti-syntaxin-8 (110083, Synaptic Systems), rabbit anti-syntaxin-12/13 (110133, Synaptic Systems), rabbit anti-syntaxin-16 (110162, Synaptic Systems), rabbit anti-SCAMP1 (PA1-739, Thermo Fisher Scientific), mouse anti-V-ATPase (149011, Synaptic Systems), mouse anti-SV2B (119111, Synaptic Systems), rabbit anti-Vti1A (165002, Synaptic Systems), rabbit anti-PKC alpha [Y124] (ab32376, Abcam), rabbit anti-YKT6 (ab236583, Abcam), rabbit anti-VAP-A (249002, Synaptic Systems), mouse anti-VCP (MA3-004, Thermo Fisher Scientific), mouse anti-otoferlin [13A9] (ab53233, Abcam), rabbit anti-otoferlin (178003, Synaptic Systems), rabbit anti-VGluT3 (135203, Synaptic Systems), guinea pig anti-VGluT3 (135204, Synaptic Systems), mouse anti-synapsin-1 (106001, Synaptic Systems), goat IgG anti-CtBP2 [E−16] (sc-5967, Santa Cruz Biotechnology), rabbit anti-Munc18-1 (116002, Synaptic Systems), rabbit anti-Munc18-2 (116102, Synaptic Systems), rabbit anti-Munc18-3 (116202, Synaptic Systems), and mouse anti-VAMP-2 (104211, Synaptic Systems). The following secondary antibodies were used: Alexa Fluor 405-conjugated donkey anti-mouse IgG (ab175658, Abcam), DyLight 405-conjugated donkey anti-guinea pig IgG (706-475-148, Jackson ImmunoResearch), MFP 488-conjugated donkey anti-goat IgG (MFP-A1055, MoBiTec), Alexa Fluor 594-conjugated donkey anti-mouse IgG (A21203, Thermo Fisher Scientific), Alexa Fluor 594-conjugated donkey anti-rabbit IgG (A21207, Thermo Fisher Scientific), Alexa Fluor 647-conjugated donkey anti-mouse IgG (A31571, Thermo Fisher Scientific), Alexa Fluor 647-conjugated donkey anti-rabbit IgG (A31573, Thermo Fisher Scientific), Alexa Fluor 647-conjugated donkey anti-goat IgG (A21447, Thermo Fisher Scientific), Alexa Fluor 488-conjugated goat anti-rabbit IgG (A11008, Invitrogen), and Alexa Fluor 568-conjugated goat anti-mouse IgG (A11004, Life technologies).

High magnification confocal images were acquired using a laser scanning confocal microscope Zeiss LSM780 (Carl Zeiss AG, Oberkochen, Germany) with a 40× Oil Plan-Apochromat objective (1.4 NA) or using a Leica TCS SP5 (Leica Microsystems GmbH, Wetzlar, Germany) with a 63× glycerol-immersion objective (1.3 NA). Low-magnification images (supplemental Fig. S1*A*) were acquired with the Leica TCS SP5 equipped with a 10× air objective (0.4 NA).

Maximum intensity projections of optical confocal sections and single-stack images were generated using Fiji (93) (https://fiji.sc/) and assembled for display in Adobe Illustrator (Adobe Systems). Color-coded 2D images were constructed in Fiji as 16-bit grayscale images to which the given color lookup table was applied.

### Auditory Brain Stem Responses (ABR)

Animals were anesthetized intraperitoneally with a combination of ketamine (125 mg/kg) and xylazine (2.5 mg/kg). The heart rate was constantly monitored to control the depth of anesthesia. The core temperature was maintained at 37°C using a rectal temperature-controlled heat blanket (Hugo Sachs Elektronik–Harvard Apparatus). For stimulus generation, presentation, and data acquisition, we used the TDT II or III Systems (Tucker Davis Technologies) run by BioSig32 software (Tucker Davis Technologies). Sound pressure levels (SPL) are provided in decibels SPL root mean square (RMS) (tonal stimuli) or decibels SPL peak equivalent (clicks) and were calibrated using a 1⁄4 inch Brüel and Kjær microphone (model 4939). Tone bursts (4/8/12/16/24/32 kHz, 10 ms plateau, 1 ms cos^2^ rise/fall) or clicks of 0.03 ms were presented at 20 Hz in the free field ipsilaterally using a JBL 2402 speaker. The difference potential between vertex and mastoid subdermal needles was amplified (50,000 times), filtered (low pass, 4 kHz; high pass, 100 Hz) and sampled at a rate of 50 kHz for 20 ms, 2 × 2000 times, to obtain two mean ABRs for each sound intensity. Hearing threshold was determined with 10 dB precision as the lowest stimulus intensity that evoked a reproducible response waveform in both traces by visual inspection.

### Patch-Clamp Recordings

Perforated patch-clamp recordings were performed as previously described (94). Briefly, the apical coils of the organ of Corti were dissected in HEPES-buffered Hank’s balanced salt solution containing (in mM): 5.36 KCl, 141.7 NaCl, one MgCl_2_, 0.5 MgSO_4_, 10 HEPES, 2.3 L-glutamin,11.1 D-glucose at pH 7.2. Recordings were performed in an extracellular solution containing (in mM): 105 NaCl, 35 tetraethylammonium (TEA)-Cl, 5 4-aminopyridine, one CsCl, 2.8 KCl, two CaCl_2_, one MgCl_2_, 10 HEPES, 11.1 D-glucose at pH 7.2. The pipette solution contained (in mM): 130 Cs-gluconate, 10 HEPES, 10 TEA-Cl, 10 4-aminopyridine, one MgCl_2,_ and 300 μg/ml amphotericin B at pH 7.2. An EPC-9 amplifier (HEKA) controlled by Pulse software (HEKA) was used for measurements. All voltages were corrected for liquid junction potentials. Currents were leak-corrected using a p/4 protocol. IHCs were stimulated by depolarizations of different durations to −14 mV at intervals of 30 to 60 s. Exocytic membrane capacitance increments (ΔC_m_) were measured as described previously (2), averaging 400 ms of capacitance data before and after (skipping the first 100 ms) the depolarization pulse. Mean ΔC_m_ and Ca^2+^ current estimates present grand averages calculated from the mean estimates of individual IHCs to avoid the dominance of IHCs contributing more sweeps.

## Results

### Establishing a Workflow to Isolate VGluT3-Positive Vesicular Organelles from Mouse Cochleae

**Fig. 2:**
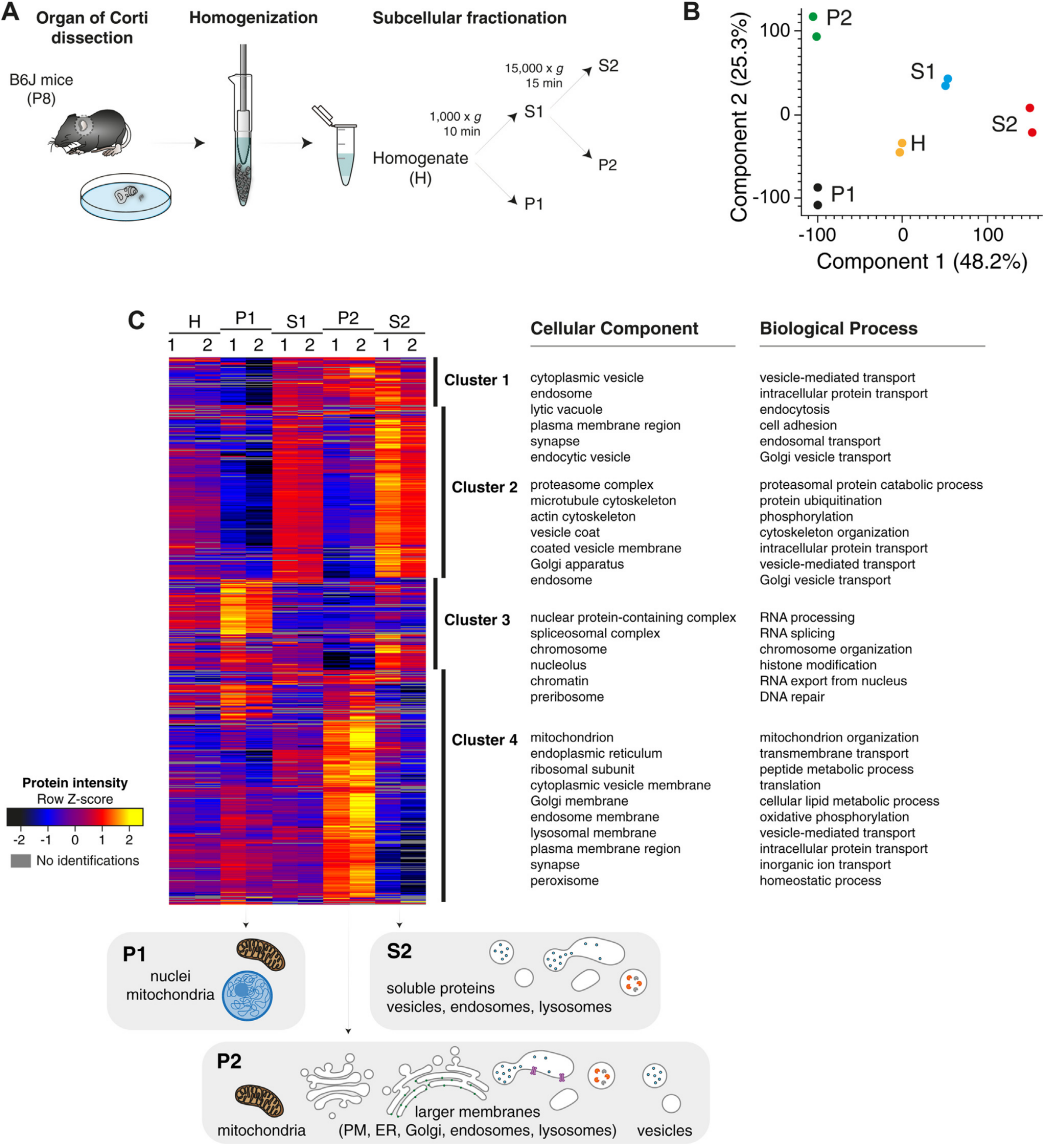
**Subcellular fractionation of P8 murine organs of Corti led to the isolation of a fraction rich in small cellular components involved in vesicle-mediated transport**. *A*, subcellular fractionation workflow. Organs of Corti of immature wild-type mice at P8 (before hearing onset) were explanted, homogenized, and fractionated by differential centrifugation. *B*, principal component analysis (PCA). The proteome of all fractions measured in duplicate segregated into five major fractions based on components 1 and 2, which account for 48.2% and 25.3% of variability, respectively. *C*, heatmap of z-scored log_2_-transformed iBAQ intensities of the differentially expressed proteins (*rows*) across fractions (*columns*) after unsupervised hierarchical clustering analysis (*yellow* indicates higher abundance and *black lower* abundance). Proteins are segregated into four clusters. The top categorical GOCC and GOBP annotations enriched for each cluster are shown (enrichment analysis performed with ShinyGO tool (86) using a Fisher’s exact test with FDR correction (*p* < 0.05)). In P1, nuclear (cluster 3) and mitochondrial (cluster 4) proteins were enriched. Proteins associated with membranes and membrane-bounded organelles and vesicles were enriched in S1. Centrifugation of S1 led to the pelleting (P2) of mitochondrial and larger membrane components (clusters 1 and 4) such as PM, Golgi, ER, and endolysosomal components. Vesicles, endolysosomal, and cytosolic components remained in the supernatant (S2) (clusters 1 and 2). S, supernatant; P, pellet; PM, plasma membrane; ER, endoplasmic reticulum; GOCC, Gene Ontology Cellular Component; GOBP, Gene Ontology Biological Process. Source data are available online for this figure (supplemental Table S1).

We set out to develop a protocol for the enrichment of mouse IHC vesicular and endosomal-like structures with a degree of purity adequate for downstream proteomic analysis (Fig. 1). We first optimized a procedure for subcellular fractionation of the organ of Corti based on protocols established for the brain (68, 69, 70, 71, 72, 73, 74). Given the need for large amounts of starting material, previous studies used intact cochleae (14, 43). We introduced a small but important variation to this procedure by dissecting the organ of Corti, the sensory epithelium where IHCs are lodged, after isolating the cochlea (Fig. 1*A*). This first purification step removes most of the cochlear bone and other tissues that could interfere with subsequent sample processing. Given the scarcity of cochlea-derived material, we used only pre-hearing mice (at P8) for optimization and validation of the subcellular fractionation protocol in order to fasten and ease the dissection step, as calcification of the cochlea is incomplete at this developmental stage (76). Approximately 150 organs of Corti from pre-hearing mice were acutely dissected, homogenized, and fractionated by differential centrifugation (Fig. 2*A*), with five fractions resulting from this procedure: homogenate (H), supernatant 1 (S1), pellet (P1), supernatant 2 (S2), and pellet 2 (P2). All fractions were in-gel digested and subjected to high-resolution LC-MS/MS to assess their protein content.

**Fig. 3:**
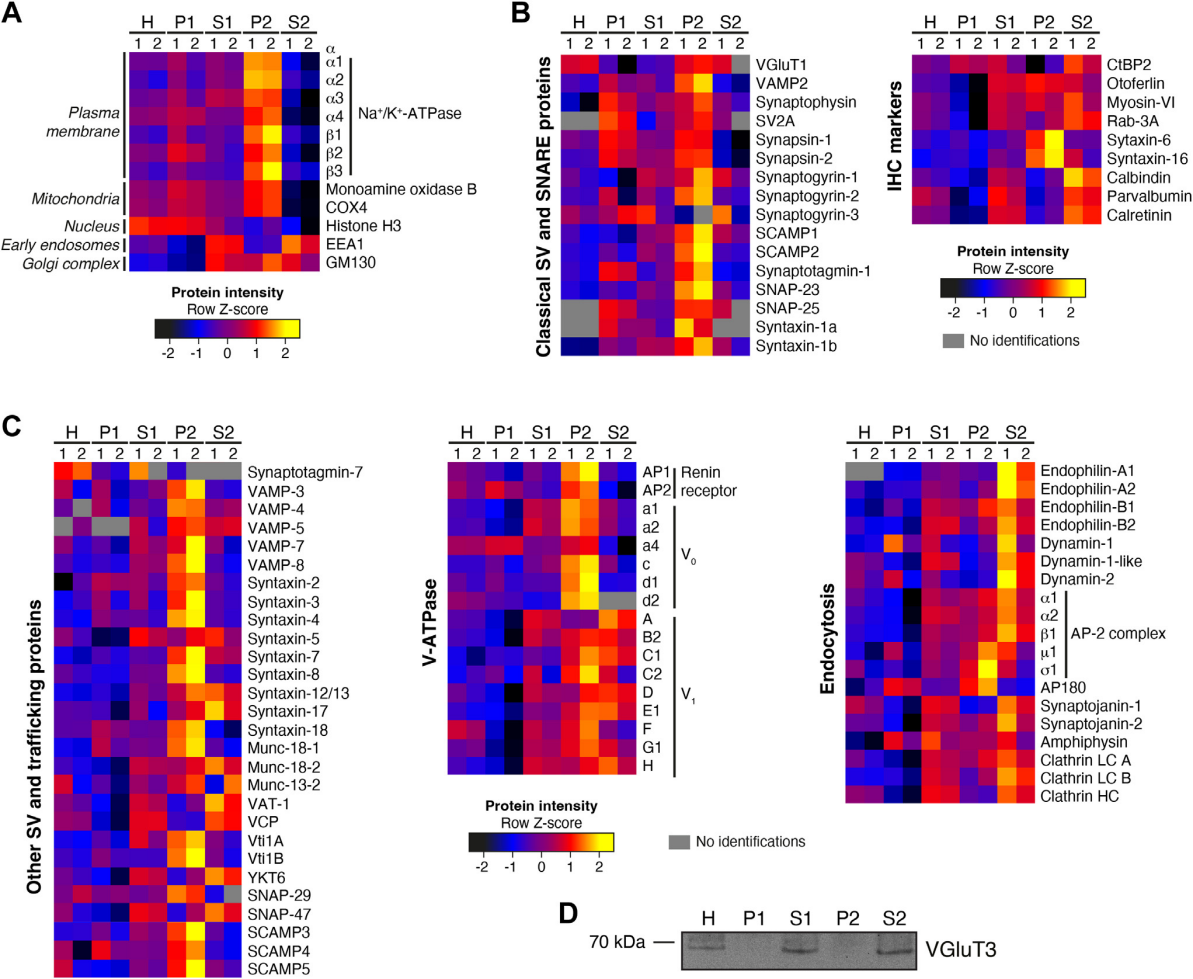
**Comparative analysis of P8 murine organ of Corti subcellular fractions at a molecular level led to the differential enrichment of both organelle and cell marker proteins in distinct fractions, and to the isolation of an IHC-rich trafficking organelles subfraction**. *A–C*, expanded cluster display of heatmap displayed in Figure 2*C*, highlighting the abundance of different proteins across different fractions (heat map of z-scored log_2_-transformed iBAQ intensities after unsupervised hierarchical clustering analysis; *yellow* indicates higher abundance and *black lower* abundance). *A*, plasma membrane and mitochondria proteins were enriched in P2, nuclear components in P1, endosomal proteins distributed to P2 and S2, Golgi proteins distributed across S1, P2, and S2. *B*, classical neuronal SV, and SNARE proteins were enriched in P2; IHC markers spread across P2 and S2 but mostly enriched in S2. *C*, other SV, trafficking, and endocytic proteins spread across P2 and S2. *D*, VGluT3 expression across subfractions monitored by immunoblotting. VGluT3 was enriched in S2 and not in P2. S, supernatant; P, pellet; SV, synaptic vesicle; IHC, inner hair cell. Source data are available online for this figure (supplemental Table S1).

Combined analysis of all fractions using the MaxQuant software (81, 82) and applying a peptide and protein false discovery rate (FDR) of 1% led to the identification of a total of 8158 proteins across all fractions (supplemental Table S1). To quantify the landscape of each fraction’s proteome and to perform a comparative proteomic analysis across fractions, we undertook an in-depth bioinformatic analysis using the Perseus software (85). Principal component analysis (PCA) revealed that samples clustered by fraction (Fig. 2*B*). To assign specific subcellular compartments to distinct subfractions, we performed an unsupervised clustering analysis followed by a cluster-specific Gene Ontology (GO)-based enrichment analysis using GO Cellular Component (CC) and GO Biological Process (BP) terms. Proteins are segregated into four major clusters, with approximately 150 to 400 annotation terms assigned per cluster (Fig. 2*C* and supplemental Table S1). Top cellular components and biological functions enriched in P1 included nuclear and mitochondrial proteins (clusters 3 and 4, respectively) (*e.g.*, histone H3, monoamine oxidase B, and COX4; see Fig. 3*A*). In S1, we observed an enrichment of proteins associated with membranes and membrane-bounded organelles and vesicles, which after high-speed centrifugation led to the pelleting (P2) of mitochondrial and larger membrane components (clusters 1 and 4) such as plasma membrane (PM), Golgi, endoplasmic reticulum (ER), and endolysosomal proteins (*e.g.*, Na^+^/K^+^-ATPase, monoamine oxidase B, COX4, and GM130; see Fig. 3*A*), whereas vesicles, endolysosomal, and cytosolic components remained in the supernatant (S2) (clusters 1 and 2). Based on classical fractionation protocols, we expected larger membrane components to distribute to P2 and smaller vesicles to remain in S2. However, vesicles and endosomal-like structures associated with vesicle-mediated transport and endocytosis are distributed also to S2 (cluster 1) (*e.g.*, SV and SNARE proteins, trafficking proteins, V-ATPase subunits, endocytosis proteins; see Fig. 3, *B* and *C*).

Analysis of the enrichment pattern of synaptic and trafficking proteins across fractions at P8 led to the identification of distinct profiles in P2 and S2 (Fig. 3, *B* and *C*). Classical neuronal SV and SNARE proteins like VGluT1, synapsin-1, SNAP-25, synaptotagmin-1, synaptobrevin/VAMP-2, and syntaxin-1, were specifically enriched in P2 (Fig. 3*B*). This suggests that conventional presynaptic terminals of efferent olivocochlear neurons majorly contributed to P2. Conversely, proteins known to be IHC markers, albeit in some cases also present in P2, were enriched in S2, *e.g.*, VGluT3, otoferlin, RIBEYE/CtBP2, Rab-3A, myosin-VI, and the Ca^2+^ buffering proteins calbindin, parvalbumin, and calretinin (Fig. 3*B*, right panel). The presence of otoferlin also in P2 is consistent with its plasma membrane localization and with the aforementioned allocation of plasma membrane components to this fraction. Endocytosis-related proteins were segregated to both P2 and S2 but were mostly enriched in S2 (Fig. 3*C*, right panel). Although the IHC-specific vesicular glutamate transporter VGluT3 was successfully detected by immunoblotting (Fig. 3*D*), no VGluT3 peptides were identified by LC-MS in any subcellular fraction, most likely due to low abundance of the protein in the organ of Corti, high composition complexity of the S2 fraction and/or due to poor fragmentation also reported for other highly hydrophobic and glycosylated integral proteins (67, 95). In fact, Hickox *et al.* were also unable to identify VGluT3 at P4-7 in the cochlea (67), and similar observations were described for the SV transporters VAChT and VMAT2 in the brain (95).

In summary, in the first low-speed centrifugation step, nuclei and other debris are pelleted, and in a second high-speed centrifugation step using the supernatant of the previous centrifugation, components of afferent (postsynaptic) and efferent nerve terminals of SGNs are pelleted while IHC constituents remain in the supernatant (fraction S2). Additional subsequent ultracentrifugation steps were attempted to further clear S2 from larger components (including potential contamination by afferent and efferent nerve terminals) in preparation for further enrichment of IHC-specific organelles by immunoisolation (where unspecific adsorption to beads often results in false positive identifications), but this resulted in significant losses (owing to the very much limited amount of starting material already obtained from a large number of organs of Corti). Based on our subcellular prefractionation, although we observed a certain loss in protein components, we applied an immunoisolation-based enrichment strategy for final purification where we used the IHC-specific expression of VGluT3 in the organ of Corti to isolate IHC-specific trafficking organelles from S2. Immunoisolation techniques have been successfully applied to obtain VGluT-specific SV subpopulations of high purity from the brain (71, 72, 96). Previous studies in IHCs have used antibodies directed against otoferlin (43, 55, 56), a widely distributed protein within IHCs (36, 97). Although we could have potentially profited from those efforts, we reasoned that targeting otoferlin would not be a good strategy, as (i) otoferlin is widely distributed in the organ of Corti, being expressed not only in IHCs but also in immature outer hair cells (OHCs) and mature OHCs of the cochlear apex (36, 98); (ii) otoferlin is abundant at the plasma membrane in IHCs (36, 42, 97); and (iii) otoferlin is also present on SVs ((36), but see (42)). Instead, we capitalized on the largely IHC-specific expression of VGluT3 within the organ of Corti (supplemental Fig. S1) where it is found not only in SVs but also, similarly to otoferlin, in other larger vesicular structures and endosomes (42, 52, 97, 99).

Using the S2 fraction as starting material, we optimized an immunobead-based isolation procedure using an antibody against VGluT3 to selectively isolate membrane vesicles containing VGluT3. For this, we coupled a monoclonal antibody against VGluT3 to magnetic beads onto which the subcellular fraction S2 was added (Fig. 1*B*). To control for unspecific binding, parallel incubations were performed with beads coupled to a control sheep IgG antibody. Immunoblot analysis of VGluT3-coupled immunobeads revealed specific binding of VGluT3-positive organelles (supplemental Fig. S2) as compared to the control, demonstrating the suitability of the beads-coupled VGluT3 antibody for enrichment of VGluT3-containing membrane vesicles in S2.

### Proteome Comparison of VGluT3-Containing Organelles Before and After Hearing Onset

**Fig. 4:**
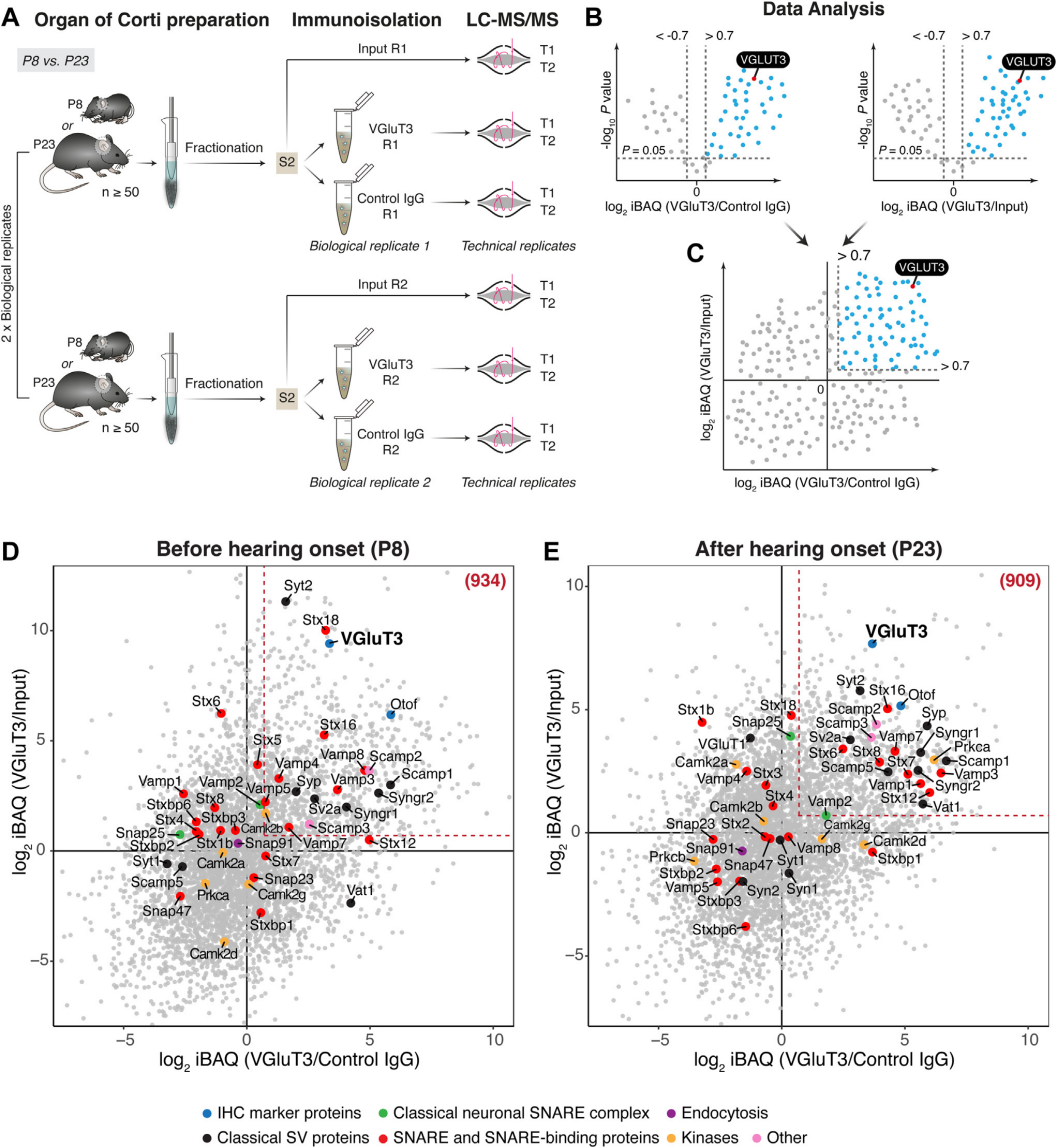
**MS-based comparative enrichment analysis of VGluT3 immunoisolates’ proteome reveals age-dependent changes in the expression of synaptic and trafficking proteins**. *A*, schematic representation of the immunoisolation approach to isolate IHC VGluT3-positive vesicular structures from immature (P8) and mature (P23) organs of Corti. S2 fraction was prepared as described in Figure 1 from ∼100 organs of Corti (per biological replicate) and used as starting material in VGluT3-and control IgG-specific immunoisolations. Two technical replicates (indicated as T1 and T2) from two independent immunoisolation procedures (biological replicates R1 and R2) were measured. *B* and *C*, approach used for the analysis of the MS data. *B*, protein enrichment was assessed by comparing VGluT3 immunoisolates with both control IgG immunoisolates and input S2 samples. The –log_10_ adjusted *p* value was plotted against the log_2_ iBAQ fold change of VGluT3 over control (IgG or input), with a significant *t* test FDR threshold of 5% and S_0_ = 0. *C*, to visualize protein enrichment in VGluT3 immunoisolates as compared to both control IgG and input S2, log_2_ iBAQ fold change VGluT3/Control IgG vs. log_2_ iBAQ fold change VGluT3/Input was plotted; proteins in the upper right quadrant were enriched in VGluT3 immunoisolates – a threshold of log_2_ iBAQ fold difference >0.7 was set to consider only proteins with at least 1.5-fold enrichment. *D* and *E*, Scatter plots showing differential enrichment of proteins in VGluT3 immunoisolates when compared to both control IgG and inputs at P8 (*D*) and P23 (*E*); displayed are IHC marker proteins VGluT3 and otoferlin, classical SV proteins, SNAREs, SNARE-binding proteins and other proteins. Numbers in parenthesis refer to the total number of significantly enriched proteins in VGluT3 immunoisolates over control IgG and Input (>1.5-fold enrichment). Gene names are displayed. *Stx12* gene annotated in UniProt refers to syntaxin-12/13 protein (syntaxin-12 and syntaxin-13 are the same protein; syntaxin-13 is the accepted term by the scientific community, but is still annotated in most databases as syntaxin-12). See also supplemental Fig. S3 for individual volcano plots. Source data are available for this figure (supplemental Table S2). IHC, inner hair cell; MS, mass spectrometry; SV, synaptic vesicle.

Having demonstrated the capability to finally enrich for VGluT3-containing membrane vesicles from the subcellular fraction S2, we compared the proteomes of VGluT3-containing membrane vesicles isolated from organs of Corti at P8 and P23, *i.e.*, before (pre-) and after (post-) hearing onset, respectively. Here, we aimed not only for an inventory of the VGluT3-specific proteome of the organ of Corti but, importantly, to assess potential developmental changes upon hearing onset at the protein expression level. To this end, we compared VGluT3-immunoisolates with both control IgG immunoisolates and input (subcellular fraction S2) samples by LC-MS. Input (S2), VGluT3-immunoisolates and control IgG immunoisolates from P8 and P23 mice were subjected to in-gel trypsin digestion and analyzed by LC-MS/MS (Fig. 4*A*). Two independent biological replicates per age (derived from two independent fractionation procedures using at least 100 organs of Corti per fractionation round), with two technical replicates each, were analyzed.

Classical immune-based proteomic mass spectrometry data analysis strategies used to ascertain the specific proteome of a given immunoisolate are based on the determination of the fold enrichment of proteins in that immunoisolate (in this case, VGluT3-immunoisolate) with a control immunoisolate using an unspecific IgG. This approach can potentially lead to false positives inherent to the beads’ nature and control IgG antibody selection, aggravated in our case by the nature of the sample (membrane structures). To compensate for this, we compared VGluT3 immunoisolates to control IgG but also to input sample S2, and we considered as final hits only those proteins enriched in VGluT3 immunoisolates when compared to both control IgG and input S2 (Fig. 4, *B* and *C*). MS data analysis of input S2, VGluT3- and IgG control-immunoisolates together (peptide and protein FDR of 1%) led to the identification of a total of 4935 and 5046 protein groups in P8 and P23 datasets, respectively (supplemental Table S2). Intensity-based absolute quantification (iBAQ) (84) values were used for quantification purposes. To identify the proteins significantly enriched in VGluT3 immunoisolates as compared with control IgG immunoisolates or input S2 samples, a two-sample two-sided *t* test (5% FDR, S_0_ = 0) was performed between the groups (VGluT3 *versus* control IgG and VGluT3 *versus* input). The results of individual comparisons were represented as volcano plots (Fig. 4*B*), in which –log_10_ of the adjusted *p* value is plotted against the log_2_ iBAQ fold change between VGluT3 and control groups individually (control IgG or input). To determine the proteins enriched in VGluT3 immunoisolates when compared to both control IgG and input samples, we plotted log_2_ iBAQ fold change (VGluT3/Input) *versus* log_2_ iBAQ fold change (VGluT3/Control IgG) (Fig. 4*C*). Proteins with log_2_ fold differences >0 in both dimensions were considered enriched in VGluT3 immunoisolates over both control IgG and input (Fig. 4*C*, upper right quadrant in log_2_ iBAQ (VGluT3/Input) *versus* log_2_ iBAQ (VGluT3/Control IgG) plot). To further increase confidence in identifications, a cutoff of log_2_ fold difference of 0.7 was set to include only proteins with at least 1.5-fold enrichment (Fig. 4*C*, proteins highlighted in light blue).

Inspection of the results (Fig. 4, *D* and *E*, supplemental Figs. S3, S4, and supplemental Table S2) showed the enrichment in proteins involved in synaptic transmission and trafficking events in IHCs, *e.g.*, VGluT3, otoferlin, and myosin-VI, and the depletion in proteins of the classical neuronal SV machinery at both ages. Proteins forming the neuronal SNARE complex for exocytosis (syntaxin-1, VAMP-2, SNAP-25), the main neuronal Ca^2+^ sensor for exocytosis synaptotagmin-1, the neuronal vesicular glutamate transporter VGluT1, and the neuronal SV proteins synapsin-1 and synapsin-2 previously shown to localize to efferent presynaptic terminals in the organ of Corti, were not enriched in VGluT3 immunoisolates at either age, supporting previous studies (30, 31, 32). However, neuronal SV proteins synaptophysin and synaptotagmin-2 were enriched at both ages although previous findings reported the absence of these proteins from mature IHCs ((28, 30), but see (100)). Moreover, we note that constitutive (31) or conditional (101) genetic deletion of SNAP-25 impair Ca^2+^ influx and viability of IHCs (101), suggesting a functional IHC expression of the protein at least during development.

**Fig. 5:**
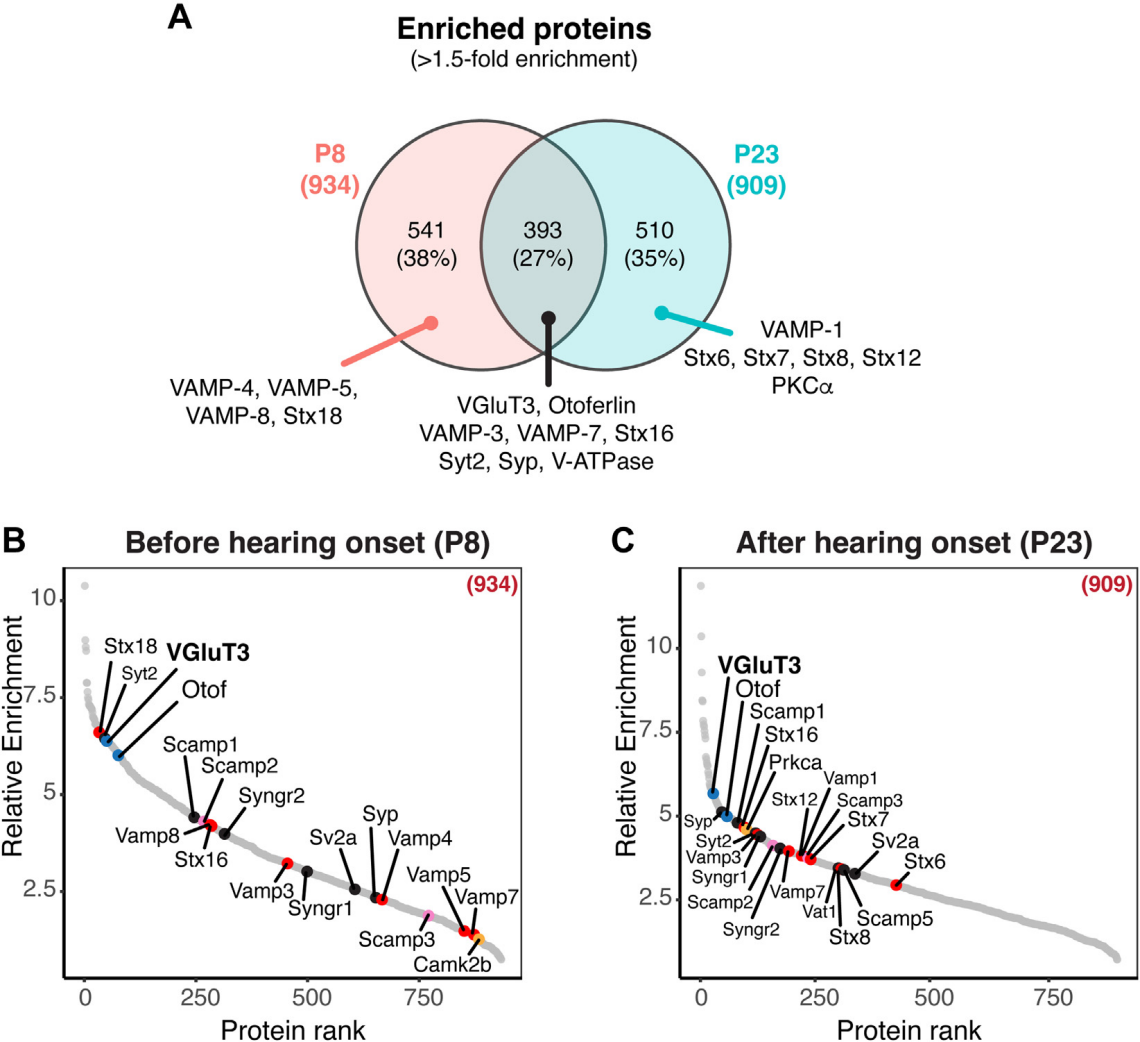
**Comparative analysis of enriched proteins in P8 and P23 VGluT3 immunoisolates revealed a small overlap in protein identifications between ages and a different relative enrichment for shared proteins**. *A*, Venn diagram showing the overlap between the significantly enriched proteins (>1.5-fold enrichment) at P8 and P23. *B* and *C*, ranking of significantly enriched proteins according to their relative enrichment in VGluT3 over Control IgG immunoisolates and input S2, before (*B*) and after (*C*) hearing onset. Relative Enrichment corresponds to the average of log_2_ iBAQ (VGluT3/Input) and log_2_ iBAQ (VGluT3/Control IgG). Displayed are only enriched proteins shown also in Figure 4, *D* and *E* (*upper right* quadrant and >1.5-fold enrichment). *Stx12* gene annotated in UniProt refers to syntaxin-12/13 protein (syntaxin-12 and syntaxin-13 are the same protein; syntaxin-13 is the accepted term by the scientific community, but is still annotated in most databases as syntaxin-12). Source data are available for this figure (supplemental Table S3).

In total, 934 and 909 proteins were unambiguously identified as enriched in P8 and P23 VGluT3 immunoisolates, respectively (>1.5-fold enrichment). A list of all significantly enriched proteins is available as supplemental Table S2. Comparative analysis revealed significant age-dependent changes, with an overlap of only 27% between P8 and P23 VGluT3-associated proteomes (Fig. 5*A* and supplemental Table S3) likely reflecting a shift in IHC protein expression upon maturation. Some of these changes involved SNARE and SNARE-binding proteins, mostly of endosomal nature. For instance, VAMP-4, VAMP-5, and VAMP-8, enriched at P8, were no longer enriched at P23 and seem to be replaced by VAMP-1 after maturation. The same pattern was observed for syntaxin-18 enriched at P8 and replaced by syntaxin-7, syntaxin-8, and syntaxin-12/13 at P23. Other proteins were enriched at both ages (VAMP-3, VAMP-7) but with differences in enrichment which can be observed by ranking the proteins by their relative enrichment in VGluT3 over control IgG immunoisolates and S2 input (*i.e.*, average of log_2_ iBAQ (VGluT3/Control IgG) and log_2_ iBAQ (VGluT3/Input)) (Fig. 5, *B* and *C*).

**Fig. 6:**
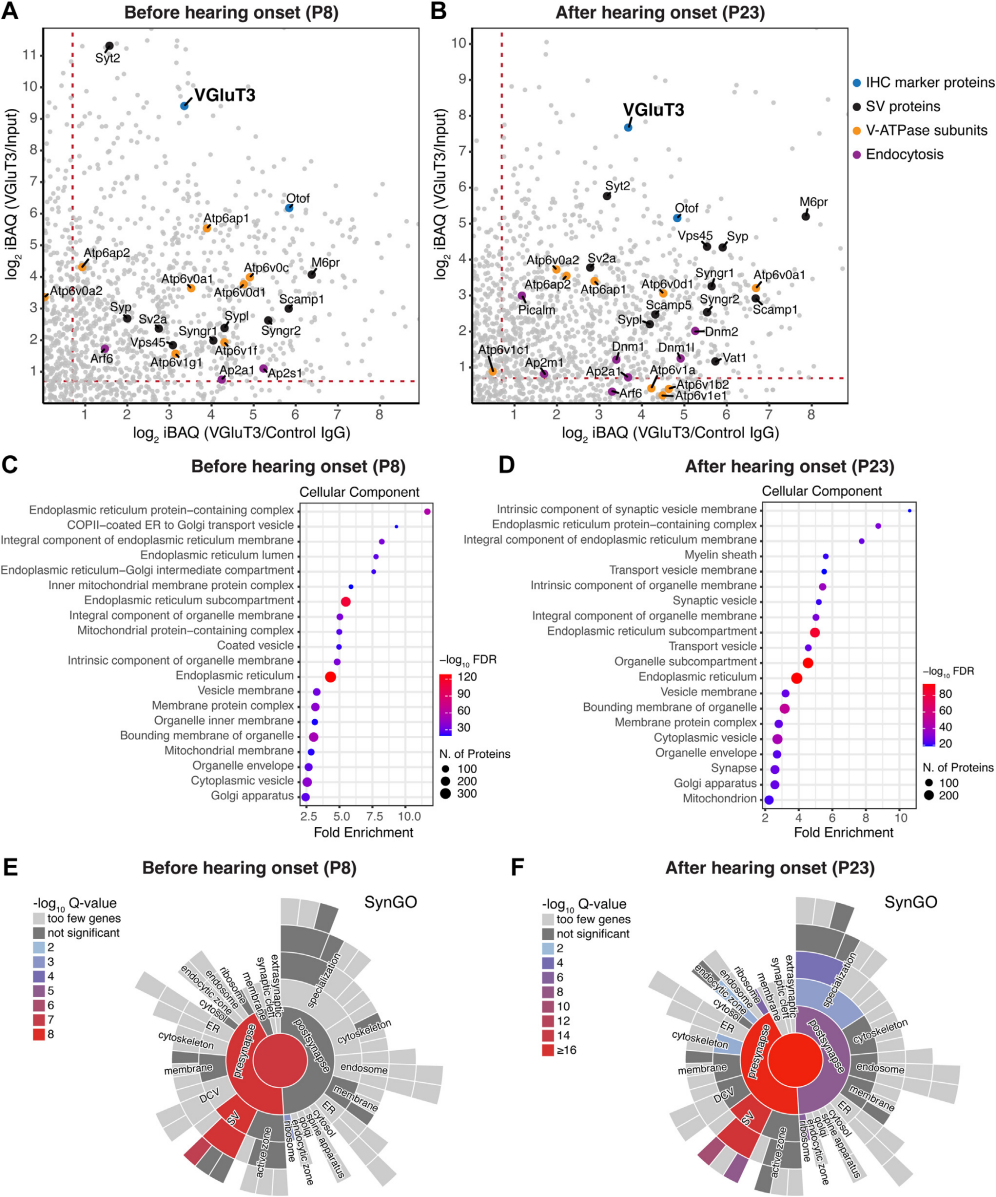
**Age-dependent changes are consistent with synapse maturation**. *A* and *B*, plots showing positively enriched proteins in VGluT3 immunoisolates when compared to both control IgG and inputs at P8 (*A*) and P23 (*B*); displayed are IHC marker proteins VGluT3 and otoferlin, and classical SV proteins; classical SV proteins were enriched at both ages but displayed a stronger enrichment at P23; recycling and endocytosis-related proteins were enriched after hearing onset only. *C* and *D*, GO cellular component enrichment analysis performed with ShinyGO (86) using a Fisher’s exact test with FDR correction (*p* < 0.05) and fold enrichment against “*Mus musculus*” background showed high enrichment of SV proteins after hearing onset; the top 20 most significant pathways are displayed; redundant pathways were filtered. *E* and *F*, mapping of enriched proteins in VGluT3 immunoisolates against the synapse-specific Synaptic Gene Ontologies (SynGO) database (87) revealed a match of 12.5% at P8 (*E*) and 16.5% at P23 (*F*) against proteins annotated in the database, showing that VGluT3-specific immunoisolated proteins are associated with synaptic function (mostly SV associated), with a slightly higher prevalence after hearing onset. Data information: Source data are available for this figure (supplemental Tables S2 and S3). IHC, inner hair cell; MS, mass spectrometry.

Some classical SV proteins were enriched at both ages although some with a higher preponderance at P23 (Figs. 4, *D* and *E* and 6, *A* and *B*). These were, *e.g.*, the vacuolar-type H^+^-ATPase (V-ATPase) complex, SCAMPs (1, 2, and 3), SV2, synaptogyrins (1 and 2), the mannose-6-phosphate receptor M6PR, and also the adaptor protein AP-2 complex involved in re-capturing SVs after exocytosis (95, 102, 103) for which functional expression in IHCs was previously demonstrated (43, 44). The vesicular monoamine transporter VMAT-1 (also known as VAT-1) (104, 105, 106), as well as recycling and endocytosis-related proteins dynamins (dynamin-1, dynamin-1-like, dynamin-2) (107), and phosphatidylinositol binding clathrin assembly protein (PICALM; also known as CALM) (108) were enriched after hearing onset only (Fig. 6, *A* and *B*). We note that functional expression of dynamin-1 has been indicated in IHCs after the onset of hearing (107, 109). A GO enrichment analysis of cellular components on both P8 and P23 datasets confirmed these results, with “intrinsic component of synaptic vesicle membrane” being the top hit GOCC term after hearing onset (Fig. 6, *C* and *D* and supplemental Table S3). A search of the final list of enriched proteins in VGluT3 immunoisolates against the synapse-specific Synaptic Gene Ontologies (SynGO) database (87) revealed a match of 12.5% for P8 and 16.5% for P23 against proteins annotated in the database (using “brain” as background), again with a higher prevalence after hearing onset, showing that VGluT3-specific immunoisolated proteins were strongly associated with synaptic function (Fig. 6, *E* and *F* and supplemental Table S3).

### Protein Composition of Mature IHC Trafficking Organelles

**Fig. 7:**
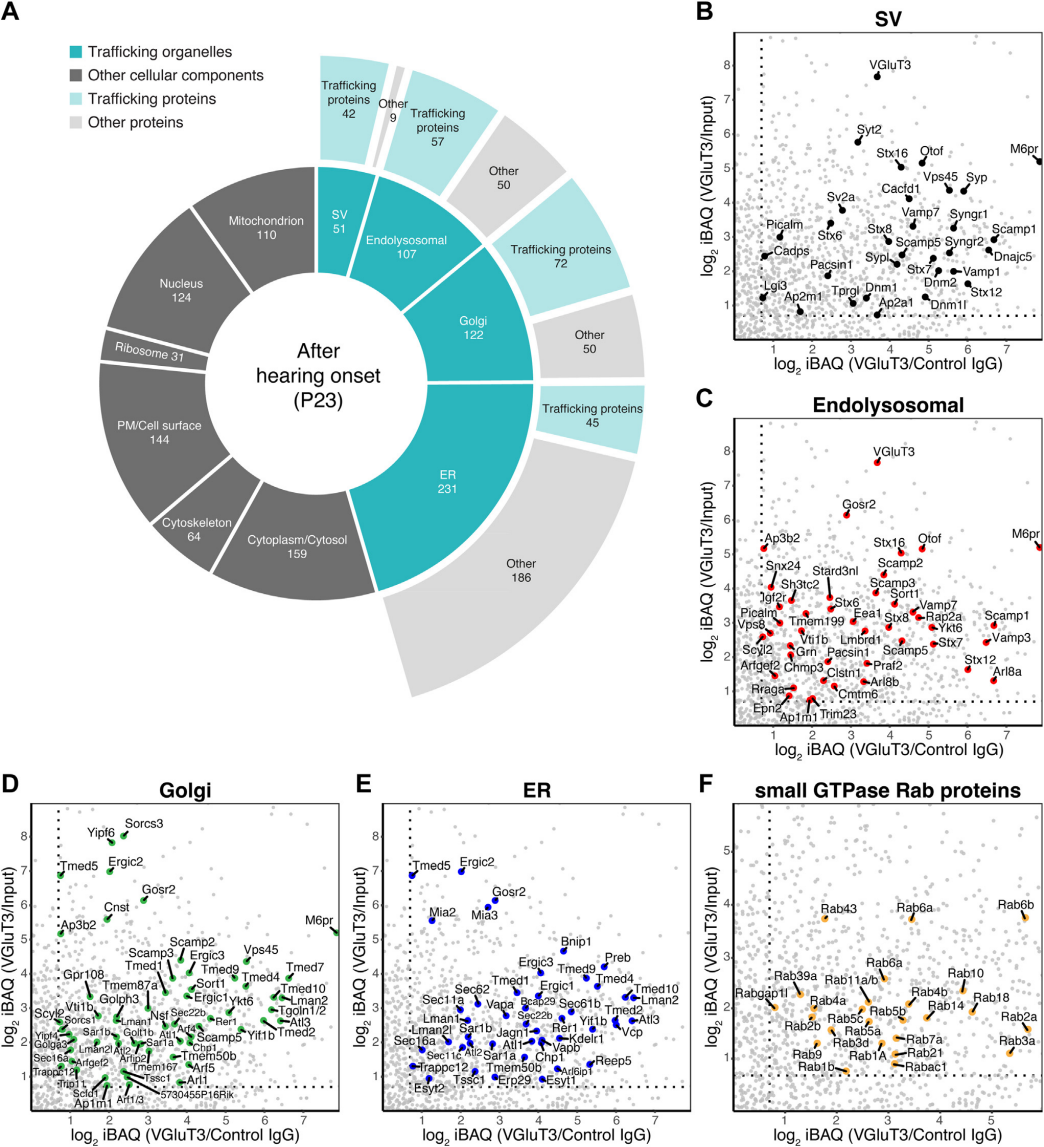
**Functional annotation of mature VGluT3-associated IHC proteome reveals a mixed SV-endosomal signature**. *A*, sunburst diagram with functional annotation of the enriched proteins in VGluT3 immunoisolates at P23. Proteins were grouped for display according to their cellular component and their involvement in trafficking events. Annotations were done manually and based on information available in several databases; proteins were grouped according to cellular component and biological function. For detailed annotations see supplemental Table S4. *B–F*, Scatter plots showing positively enriched proteins in VGluT3 immunoisolates at P23 when compared to both control IgG and input; log_2_ iBAQ fold change VGluT3/Control IgG vs. log_2_ iBAQ fold change VGluT3/Input was plotted. Displayed are proteins involved in trafficking events in different trafficking organelles (SV, endolysosomal, Golgi, and ER proteins) including SNAREs and resident proteins. Several Rab GTPase proteins, mostly of endolysosomal nature, were also enriched (*F*). Annotations were done manually and based on information available in several databases; proteins were grouped according to cellular compartment and biological function. For detailed annotation see supplemental Table S4. In (*B*–*F*) gene names are displayed. *Stx12* gene annotated in UniProt refers to syntaxin-12/13 protein (syntaxin-12 and syntaxin-13 are the same protein; syntaxin-13 is the accepted term by the scientific community but is still annotated in most databases as syntaxin-12). For ranking of proteins according to their relative enrichment, see supplemental Fig. S5. Source data are available for this figure (supplemental Table S2). ER, endoplasmic reticulum; PM, plasma membrane; SV, synaptic vesicle.

After identifying developmental changes in the VGluT3-associated proteome, we next set out to fully annotate the proteome of our P23 VGluT3 immunoisolates in order to identify proteins potentially important to trafficking and presynaptic activity after IHC maturation (supplemental Table S4, Fig. 7, and supplemental Fig. S5). We found that the P23 VGluT3 immunoisolates contained SV, endosomal, lysosomal, Golgi, ER, plasma membrane, and mitochondrial proteins. These included SNAREs and SNARE regulators, endocytosis-related proteins, proteins involved in intracellular membrane trafficking, receptors, transporters, and channels. The contribution of diverse organelles was evident by the presence of resident “marker” proteins like ER-resident proteins (*e.g.*, VAMP-associated protein B (VAP-B) and extended synaptotagmins (E-Syt1 and E-Syt2)), Golgi complex proteins (*e.g.*, GM130), and endosomal/lysosomal markers (*e.g.*, EEA1, Rab5, Rab7, LAMP-1, LAMP-2). A large number of cytoplasmic and peripheral membrane proteins was also identified, including (i) GTPases involved in trafficking, (ii) signaling proteins like kinases and phosphatases, (iii) cytoskeleton proteins, (iv) metabolic enzymes, and (v) chaperones. While some of the identified proteins might be involved in vesicle fusion and other trafficking events (*e.g.*, GTPases and cytoskeleton proteins) and be associated with membranes, others are probably contaminants. If fact, we also detected some ribosomal and nuclear proteins, together with proteins involved in RNA processing and proteasome components. Ribosomes and proteasome components are in the same size range of SVs and are expected to copurify with these. Ribosomal and nuclear proteins are known background contaminants of affinity purification due to interactions with the solid-phase support, affinity reagent, or epitope tags (110).

Of the enriched proteins a considerable fraction is known to be involved in trafficking events (Fig. 7*A*, Table 1, and supplemental Table S4). A substantial number of the identified trafficking proteins were previously identified as SV proteins in conventional synapses or were of endolysosomal nature (and predominantly endosomal) (Fig. 7, *A*–*C*). Endosomal SNAREs and SNARE-binding proteins included VAMP-3, VAMP-7, syntaxin-6, syntaxin-7, syntaxin-8, syntaxin-12/13, syntaxin-16, and Vps8, some of which are SNAREs functioning not only in early and late endosome fusion, but also in SV-endosome fusion. This SV-endosomal signature seems consistent with the needed high rates of vesicle recycling and resupply at IHC synapses (review in (9)). Syntaxins −6 and −16, and LAMP-1, however, were reported to be predominantly expressed in the apical part of the IHCs, where constitutive membrane trafficking events occur and were deemed not to be operational in synaptic transmission in these cells (97). Trafficking proteins belonging to the ER and Golgi were mostly proteins involved in membrane fusion events and trafficking between the two compartments (Fig. 7, *A*, *D*, and *E*). These include some SNAREs like SEC20 (gene *Bnip1*) and SEC22B, but also others additionally involved in endosome to Golgi trafficking like the Golgi SNAP receptor complex member two protein (GOSR2), the vesicle transport through interaction with t-SNAREs homolog 1B (Vti1B), and the synaptobrevin homolog YKT6. A multitude of small GTPase Rab proteins, often regarded as “organelle markers”, was also identified (Fig. 7*F*): these were again mostly of endolysosomal nature. Of these, Rab-3 (A and D), an SV Rab, was previously reported to be expressed in IHCs (14, 97).

**Table 1:** Summary of SV, SNARE, and other trafficking proteins enriched in VGluT3 immunoisolates at P23 and identified by MS analysis

Gene names	Protein names	Cellular compartment
IHC markers		
*Slc17a8*	Vesicular glutamate transporter 3 (VGluT3)	SV; Endolysosomal (Endosome)
*Otof*	Otoferlin	PM; SV; Endolysosomal (Endosome)
SNARE and SNARE-binding		
*Vamp1*	Vesicle-associated membrane protein 1	SV
*Vamp3*	Vesicle-associated membrane protein 3	Endolysosomal (Endosome)
*Vamp7*	Vesicle-associated membrane protein 7	SV; Endolysosomal (Endosome)
*Stx6*	Syntaxin-6	SV; Endolysosomal (Endosome)
*Stx7*	Syntaxin-7	SV; Endolysosomal (Endosome)
*Stx8*	Syntaxin-8	SV; Endolysosomal (Endosome; Lysosome)
*Stx12*	Syntaxin-12	SV; Endolysosomal (Endosome)
*Stx16*	Syntaxin-16	SV; Endolysosomal (Endosome)
*Syt2*	Synaptotagmin-2	SV
*Esyt1*	Extended synaptotagmin-1	ER
*Esyt2*	Extended synaptotagmin-2	ER
*Bnip1*	Vesicle transport protein SEC20	ER
*Sec22b*	Vesicle-trafficking protein SEC22b	ER; Golgi
*Vti1b*	Vesicle transport through interaction with t-SNAREs homolog 1B	Golgi; Endolysosomal (Endosome)
*Ykt6*	Synaptobrevin homolog YKT6	Golgi; Endolysosomal (Endosome)
*Gosr2*	Golgi SNAP receptor complex member 2	ER; Golgi; Endolysosomal (Endosome)
*Napa*	Alpha-soluble NSF attachment protein	PM
*Napb*	Beta-soluble NSF attachment protein	PM
*Napg*	Gamma-soluble NSF attachment protein	PM
*Lgi3*	Leucine-rich repeat LGI family member 3	SV
Other trafficking and SV proteins		
*Atp6ap1*	V-type proton ATPase AP1 (V-ATPase complex; Renin receptor)	SV; Endolysosomal (Lysosome; Endosome)
*Atp6ap2*	V-type proton ATPase AP2 (V-ATPase complex; Renin receptor)	SV; Endolysosomal (Lysosome; Endosome)
*Atp6v0a1*	V-type proton ATPase subunit a1 (V-ATPase complex; V0-a1)	SV; Endolysosomal (Lysosome; Endosome)
*Atp6v0a2*	V-type proton ATPase subunit a2 (V-ATPase complex; V0-a2)	SV; Endolysosomal (Lysosome; Endosome)
*Atp6v0d1*	V-type proton ATPase subunit d1 (V-ATPase complex; V0-d1)	SV; Endolysosomal (Lysosome; Endosome)
*Dnajc5*	DnaJ homolog subfamily C member 5 (CSP-α)	SV
*Scamp1*	Secretory carrier-associated membrane protein 1	SV; Endolysosomal (Endosome)
*Scamp2*	Secretory carrier-associated membrane protein 2	Golgi; Endolysosomal (Endosome)
*Scamp3*	Secretory carrier-associated membrane protein 3	Golgi; Endolysosomal (Endosome)
*Scamp5*	Secretory carrier-associated membrane protein 5	SV; Golgi; Endolysosomal (Endosome)
*Sv2a*	Synaptic vesicle glycoprotein 2A	SV
*Syngr1*	Synaptogyrin-1	SV
*Syngr2*	Synaptogyrin-2	SV
*Syp*	Synaptophysin	SV
*Sypl1*; *Sypl*	Synaptophysin-like protein 1	SV
*Vat1*	Synaptic vesicle membrane protein VAT-1 homolog (VMAT1)	SV
*Vapa*	Vesicle-associated membrane protein-associated protein A	PM; ER
*Vapb*	Vesicle-associated membrane protein-associated protein B	ER
*Vcp*	Transitional endoplasmic reticulum ATPase	ER
*Vps8*	Vacuolar protein sorting-associated protein 8 homolog	Endolysosomal (Endosome)
*Vps45*	Vacuolar protein sorting-associated protein 45	Golgi; SV
*Tprg1l*; *Tprgl*	Tumor protein p63-regulated gene 1-like protein	SV
*Wfs1*	Wolframin	ER, SV
*Nsf*	Vesicle-fusing ATPase	Golgi; PM
*M6pr*	Cation-dependent mannose-6-phosphate receptor	SV; Endolysosomal (Endosome; Lysosome); Golgi
*Cadps*	Calcium-dependent secretion activator 1	SV
*Cacfd1*	Calcium channel flower homolog	SV
*Cacna2d2*	Voltage-dependent calcium channel subunit alpha-2/delta-2; Voltage-dependent calcium channel subunit alpha-2-2; Voltage-dependent calcium channel subunit delta-2	PM
small GTPases and related		
*Rab1A*	Ras-related protein Rab-1A	Golgi
*Rab1b*	Ras-related protein Rab-1B	Endolysosomal (Lysosome)
*Rab21*	Ras-related protein Rab-21	Endolysosomal (Endosome)
*Rab2a*	Ras-related protein Rab-2A	SV; Golgi
*Rab2b*	Ras-related protein Rab-2B	Golgi
*Rab3a*	Ras-related protein Rab-3A	SV; Endolysosomal (Endosome)
*Rab3d*	Ras-related protein Rab-3D	Endolysosomal (Endosome)
*Rab4a*	Ras-related protein Rab-4A	SV; Endolysosomal (Endosome)
*Rab4b*	Ras-related protein Rab-4B	SV; Endolysosomal (Endosome)
*Rab5a*	Ras-related protein Rab-5A	SV; Endolysosomal (Endosome)
*Rab5b*	Ras-related protein Rab-5B	SV; Endolysosomal (Endosome)
*Rab5c*	Ras-related protein Rab-5C	SV; Endolysosomal (Endosome)
*Rab6a*	Ras-related protein Rab-6A	Golgi
*Rab6a*	Ras-related protein Rab-6A	Golgi
*Rab6b*	Ras-related protein Rab-6B	Golgi
*Rab7a*	Ras-related protein Rab-7a	SV; Endolysosomal (Endosome)
*Rab9a*; *Rab9*	Ras-related protein Rab-9A	Endolysosomal (Endosome)
*Rab10*	Ras-related protein Rab-10	SV; Golgi
*Rab11b*; *Rab11a*	Ras-related protein Rab-11B; Ras-related protein Rab-11A	SV; Endolysosomal (Endosome)
*Rab14*	Ras-related protein Rab-14	SV; Endolysosomal (Endosome)
*Rab18*	Ras-related protein Rab-18	Golgi
*Rab39a*	Ras-related protein Rab-39A	Golgi
*Rab43*	Ras-related protein Rab-43	Golgi
*Rabac1*	Prenylated Rab acceptor protein 1	Golgi
*Rabgap1l*	Rab GTPase-activating protein 1-like	Golgi; Endolysosomal (Endosome)
Trafficking-related kinases		
*Prkca*	Protein kinase C;Protein kinase C alpha type (PKCα)	Cytoplasm
*Pacsin1*	Protein kinase C and casein kinase substrate in neurons protein 1	PM; Endolysosomal (Endosome); SV
Endocytosis		
*Ap1m1*	AP-1 complex subunit mu-1	Golgi; Endolysosomal (Endosome)
*Ap2a1*	AP-2 complex subunit alpha-1	SV
*Ap2m1*	AP-2 complex subunit mu	SV
*Ap3b2*	AP-3 complex subunit beta-2	Golgi; Endolysosomal (Endosome)
*Dnm1*	Dynamin-1	SV
*Dnm1l*	Dynamin-1-like protein	SV
*Dnm2*	Dynamin-2	SV
*Picalm*	Phosphatidylinositol-binding clathrin assembly protein	PM; Endolysosomal (Endosome); SV
*Diap1*; *Diaph1*	Protein diaphanous homolog 1	Cytoskeleton
Inositol metabolism		
*Cdipt*	CDP-diacylglycerol--inositol 3-phosphatidyltransferase	PM; ER; Golgi
*Gpaa1*	Glycosylphosphatidylinositol anchor attachment 1 protein	Cytoplasm; ER; Mitochondrion
*Impa2*	Inositol monophosphatase 2	Cytoplasm
*Itpr1*	Inositol 1,4,5-trisphosphate receptor type 1	ER
*Mdga1*	MAM domain-containing glycosylphosphatidylinositol anchor protein 1	PM; Golgi
*Nudt11*; *Nudt10*	Diphosphoinositol polyphosphate phosphohydrolase 3-beta; Diphosphoinositol polyphosphate phosphohydrolase 3-alpha	Cytosol
*Pi4k2a*	Phosphatidylinositol 4-kinase type 2-alpha	Endolysosomal (Endosome; Lysosome); PM
*Pitpnm1*	Membrane-associated phosphatidylinositol transfer protein 1	Cytoplasm; ER; Golgi
*Plcb4*	Phospholipase C, beta 4 (Fragment)	Cytosol
*Tmem55a*	Type 2 phosphatidylinositol 4,5-bisphosphate 4-phosphatase	Endolysosomal (Endosome; Lysosome)
*Tmem55b*	Type 1 phosphatidylinositol 4,5-bisphosphate 4-phosphatase	Endolysosomal (Endosome; Lysosome)

In some cases, protein name abbreviations are shown in parentheses. The full list of proteins as well as their biological function can be found in supplemental Table S4. ER, endoplasmic reticulum; MS, mass spectrometry; PM, plasma membrane; SV, synaptic vesicle.

We note that some proteins were enriched in VGluT3 immunoisolates when compared to control IgG (enrichment factor >1.5 in VGluT3 vs. control IgG) but not in VGluT3 immunoisolates when compared to S2 input. An example is CaMKII (CaMKIIδ and γ; gene names: *Camk2g* and *Camk2d*), an SV-resident kinase in conventional synapses and proposed to regulate exocytosis in IHCs *via* phosphorylation of otoferlin (59) (Fig. 4*E* and supplemental Fig. S4).

In summary, our MS-based data of VGluT3 immunoisolated species shows a clear signature of SV and endosomal nature, revealing a unique molecular composition in IHC different from that of classical neuronal synapses.

### Comparison of the LC-MS/MS Hits to IHC and SGN Transcriptome Data

The transcriptome database UMgear.org was used to access publicly available 10× single-cell RNA sequencing datasets. We focused on the dataset by (111) providing gene expression data for P8, P12, and P20 mouse cochlea on the single-cell level as it was close to the age analyzed here. Gene expression data was assessed for IHCs and type I SGNs for the LC-MS/MS hits of the present study. For most proteins enriched in VGluT3 immunoisolates, gene expression in IHCs was validated, yet at highly variable levels (supplemental Fig. S6). Interestingly, mRNA of VGluT3 itself was detectable neither in this IHC dataset nor in another 10× single IHC RNA sequencing dataset (112), possibly due to the limited transcriptome depth of these single-cell approaches. In agreement with our enrichment data, expression of several genes in IHCs was robustly upregulated after the onset of hearing, including all proteins enriched in VGluT3 immunoisolates at P23 only (*Dnm1, Dnm1l, Dnm2, Picalm* and *Vat1*; supplemental Fig. S6*E*) but also otoferlin, synaptotagmin and Munc18 (supplemental Fig. S6*A*, but see Fig. 10). In contrast, the observed shift in synaptobrevin protein composition was not reflected by the IHC transcriptome data, where mRNA for *Vamp1, 2, 4,* *5* and *8* was increased after hearing onset (supplemental Fig. S6*C*). This might indicate a mismatch of mRNA and protein abundance that was previously indicated for neuronal SNAREs (31). Comparing IHC and SGN datasets is interesting in this context: *Vamp1* was found to be expressed in IHCs only weakly, but very abundantly in SGNs. The age-dependent changes of syntaxins in our immunofractions with initial abundance of syntaxin-18 at P8 which is replaced by syntaxin-7, -8 and -12 at P23, were not reflected in changes in the IHC transcriptome. *Stx7,* *Stx8* and *Stx12* expression was higher in SGNs than in IHCs. The enrichment observed for SCAMP1, synaptogyrin-2 (gene *Syngr2*) and M6PR in P23 immunofractions was partially reflected in the IHC transcriptome data, while expression of the respective genes in SGNs was not detectable or at a similar level like in IHC. This underlines the value of subcellular fraction proteome data as a resource for identification of functional protein units.

### Validation of LC-MS/MS Hits by Immunofluorescence Microscopy and Functional Analysis

**Fig. 8:**
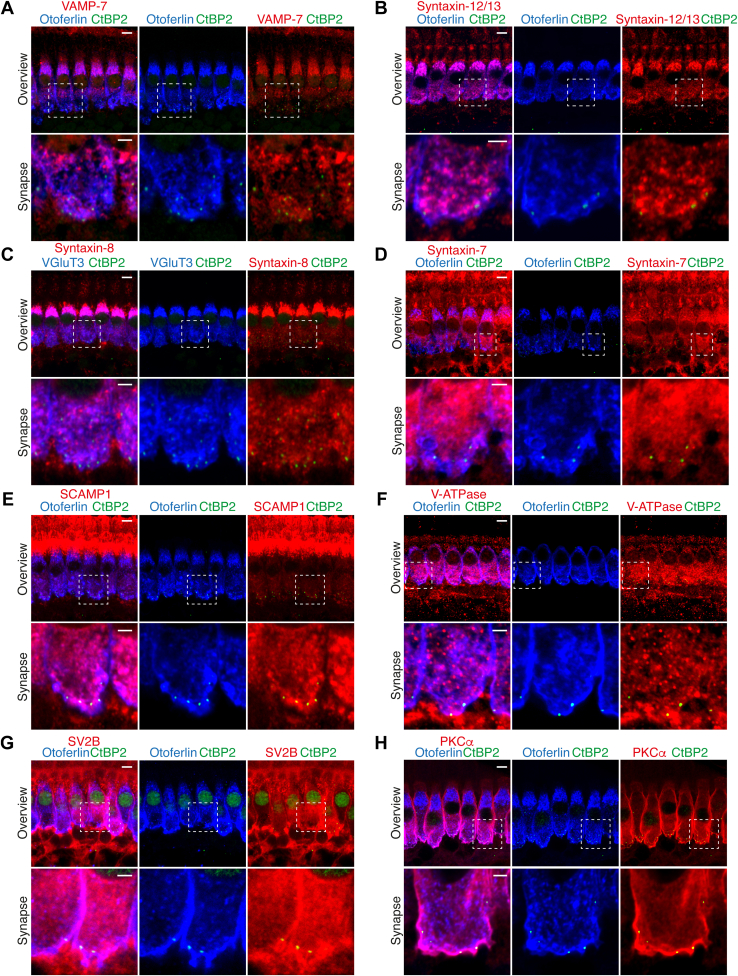
**Immunolocalization analysis of SNARE, SV, and kinase proteins in the adult organ of Corti**. *A–D*, VAMP-7 (*A*), syntaxin-12/13 (*B*), syntaxin-8 (*C*), and syntaxin-7 (*D*), all highly enriched in our MS experiments, are expressed in IHCs. *E–H*, some classical neuronal SV proteins SCAMP1 (*E*), V-ATPase (*F*), and SV2B (*G*), and the kinase PKCα (*H*), all enriched in our MS experiments, are expressed in IHCs. Note that all proteins localize to the basolateral region of the IHCs, although in some cases the protein is also expressed in supporting cells and/or in afferent (postsynaptic) and efferent fibers of SGNs. Images correspond to high magnification views of representative P15–25 IHCs immunolabeled with antibodies against the candidate proteins (*red*), the ribbon marker CtBP2/RIBEYE (*green*), and the IHC marker otoferlin (*blue*). The upper panels show overviews of representative IHCs, displaying maximum intensity projections of 5 to 10 confocal optical sections through the longitudinal axis of the IHCs (scale bars: 5 μm). The bottom panels show a zoom into the synaptic area, displaying single confocal optical sections through the longitudinal axis of a single IHCs at the basal region (scale bars: 2 μm). IHC, inner hair cell; MS, mass spectrometry; SGN, spiral ganglion neuron.

**Fig. 9:**
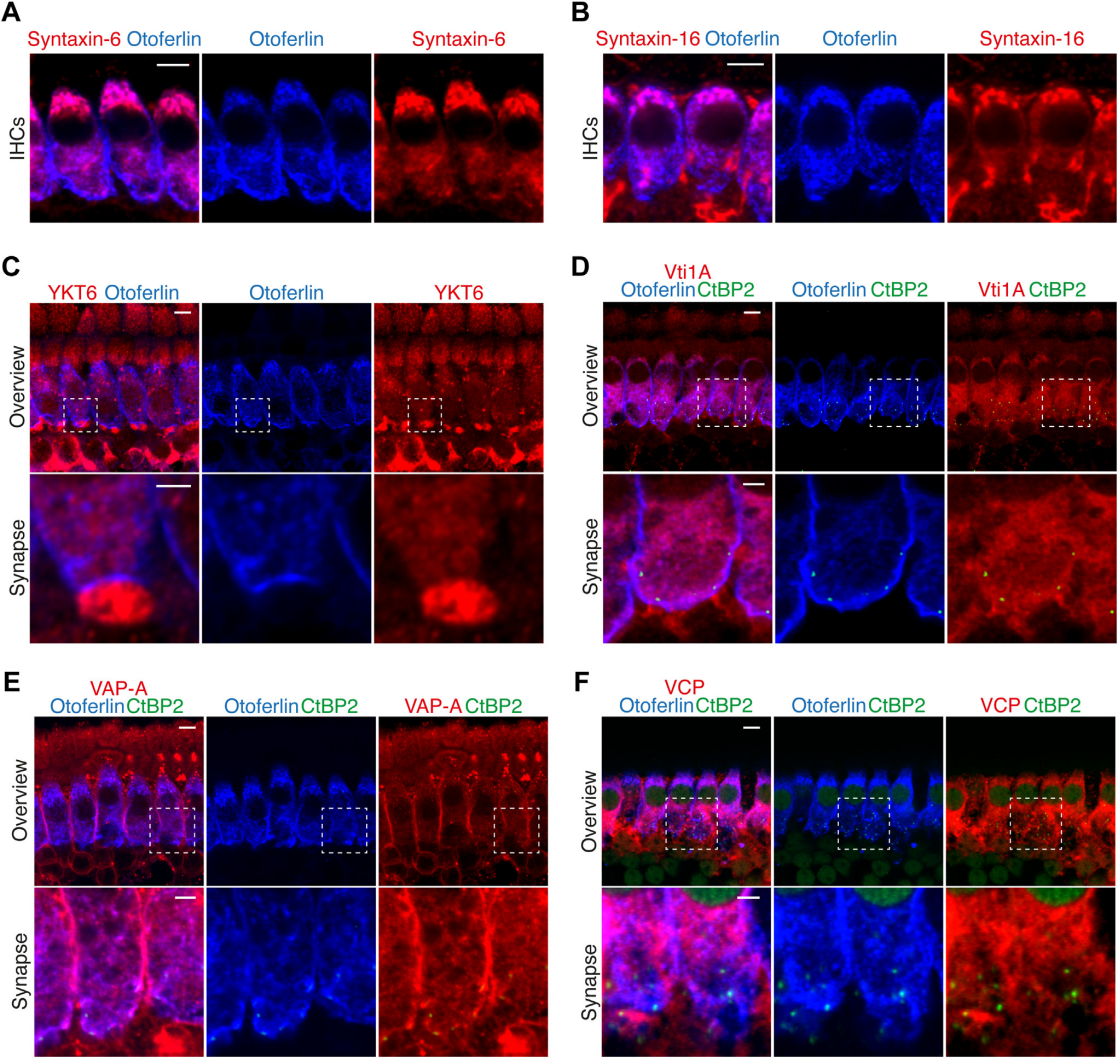
**Immunolocalization analysis of trafficking proteins in the adult organ of Corti**. *A* and *B*, Syntaxin-6 (*A*) and syntaxin-16 (*B*), enriched in our MS experiments, were previously shown to be expressed in IHCs. *C–F*, The Golgi and endosomal markers YKT6 (*C*) and VtiA (*D*), the ER and presynaptic plasma membrane protein VAP-A (*E*), the ER protein VCP (*F*) are expressed in IHCs. Note that all proteins localize to the basolateral region of the IHCs, although in some cases the protein is also expressed in afferent (postsynaptic) and efferent fibers of SGNs. Images correspond to high magnification views of representative P15–25 IHCs immunolabeled with antibodies against the candidate proteins (*red*), the ribbon marker CtBP2/RIBEYE (*green*), and the IHC marker otoferlin (*blue*). In (*C–F*), the upper panels show overviews of representative IHCs, displaying maximum intensity projections of 5 to 10 confocal optical sections through the longitudinal axis of the IHCs (scale bars: 5 μm). The bottom panels show a zoom into the synaptic area, displaying single confocal optical sections through the longitudinal axis of a single IHC at the basal region (scale bars: 2 μm). IHC, inner hair cell; SGN, spiral ganglion neuron.

We next validated a selection of the MS hits by evaluating their presence in mature IHCs *via* immunohistochemistry and confocal microscopy (Figs. 8, 9, and supplemental Figs. S7–S9). Whole-mount explants of apical turns of organs of Corti of P15–25 mice were immunolabelled using antibodies against the candidate proteins. No significant differences were observed between P15 and P25, and the results presented here are representative of three to five independent stainings. We specifically focused on proteins involved in exocytic, endocytic, and other vesicular trafficking events, that is, SV proteins, SNARE, and SNARE-binding proteins. Colabeling with antibodies against the IHC markers otoferlin and VGluT3 and against the ribbon marker RIBEYE/CtBP2, allowed to test the localization of immunofluorescence within IHCs and at their ribbon-type active zone. We note that the immunofluorescence of otoferlin and VGluT3 is specific to IHCs, where both signals largely overlap (*e.g.* (39, 42, 52, 113)) and hence otoferlin was primarily used as an IHC marker in the present study. In some cases, synapsin-1 or VAMP-2 were used to label efferent nerve terminals. We note that this study attempted to validate positive immunofluorescence with the respective knockout tissues only in the case of Munc18-1.

Of all VAMPs enriched in our MS data, we found VAMP-7 signal in mature IHCs as well as in other cell types (Fig. 8*A*). In IHCs, VAMP-7 signal was observed at both the apical compartment and the basolateral synaptic pole.

Syntaxin-6, syntaxin-7, syntaxin-8, syntaxin-12/13, and syntaxin-16 were positive hits in our MS data. We reexamined the presence of syntaxin-6 and syntaxin-16 in the mature organ of Corti (Fig. 9, *A* and *B*). As previously reported, their immunofluorescence was predominant in the apical part of the cells where constitutive membrane trafficking events occur, and does not colocalize with otoferlin, hence their direct involvement in SV exocytosis and synaptic transmission is unlikely (97). Our immunostainings were also positive for syntaxin-7, syntaxin-8, and syntaxin-12/13 in mature IHCs (Fig. 8, *B*–*D*), in support of our MS data. Syntaxin-7 seems to localize to IHCs as well as many other cell types in the organ of Corti. The syntaxin-7 signal in IHCs is distributed across the whole cell. Syntaxin-8 signal was observed throughout the IHC but with a stronger intensity at the apex. Syntaxin-12/13 signal, while present across the whole IHC, appears to concentrate near the basolateral membrane and overlaps well with that of otoferlin, which might suggest a role in the SV cycle in IHCs.

Consistent with our proteomics results, immunofluorescence analysis also indicated the presence of several SV proteins in mature IHCs: SCAMP1, V-ATPase, and SV2 (Fig. 8, *E*–*G*). In contrast, we did not find immunofluorescent signal in IHCs for synaptophysin (supplemental Fig. S7*A*), one of the top hits in our mass spectrometry analysis, which is consistent with previous studies (28) and suggests that the immunoisolate contains some efferent neural contamination. PKCα, a kinase involved in synaptic transmission and vesicle recycling events in neurons (114, 115, 116, 117, 118, 119), passed the cutoff and was highly enriched in our MS data at P23. PKCα immunostaining supports expression in mature IHCs and the fluorescence signal appears to overlap with that of otoferlin (Fig. 8*H*).

An interesting and highly conserved SNARE, the synaptobrevin homolog YKT6 (120), was enriched in our proteomics data, and its expression in mature IHCs was also indicated by immunostaining (Fig. 9*C*). In mammalian cells, YKT6 has been reported to localize to both cytosol and Golgi membranes, and sometimes to the perinuclear area (120, 121, 122). In neuronal cells, YKT6 localizes to compartments of unknown origin (123). In adult organs of Corti, we found YKT6 to localize to the postsynaptic boutons of SGNs and to IHCs. In IHCs, while YKT6 expression was dispersed across the cell, fluorescent puncta both above and below the nuclear area of the IHC seem to be consistent with an endosomal and/or Golgi localization. Vti1A, a Golgi and endosomal marker also enriched in our MS data, also seems to be present in IHCs according to immunolabeling (Fig. 9*D*). We could further observe the expression of the vesicle-associated membrane protein VAP-A in IHCs (Fig. 9*E*), a membrane receptor for lipid- and sterol-binding proteins which usually localizes to the ER but is also an integral component of the presynaptic membrane in neurons. VAP-A staining was observed in the organ of Corti both in IHCs and supporting cells; in IHCs it seems to distribute mainly to the plasma membrane. An antibody against the transitional endoplasmic reticulum ATPase VCP, enriched in our MS analysis, labeled IHCs and adjacent cells (likely SGN fibers) (Fig. 9*F*). While VCP immunostaining was observed throughout the IHC, a stronger signal was observed at the perinuclear region, consistent with an ER localization.

Synaptotagmin-2 was highly enriched in our VGluT3 immunoisolates at both ages. In our immunohistochemistry analysis, we observed synaptotagmin-2 signal in IHCs (supplemental Fig. S7*B*) contrasting several previous reports that could not detect its expression in mature IHCs (28, 30). However, our results seem to validate one study where synaptotagmin-2 immunolabelling was observed in adult mouse IHCs (100).

**Fig. 10:**
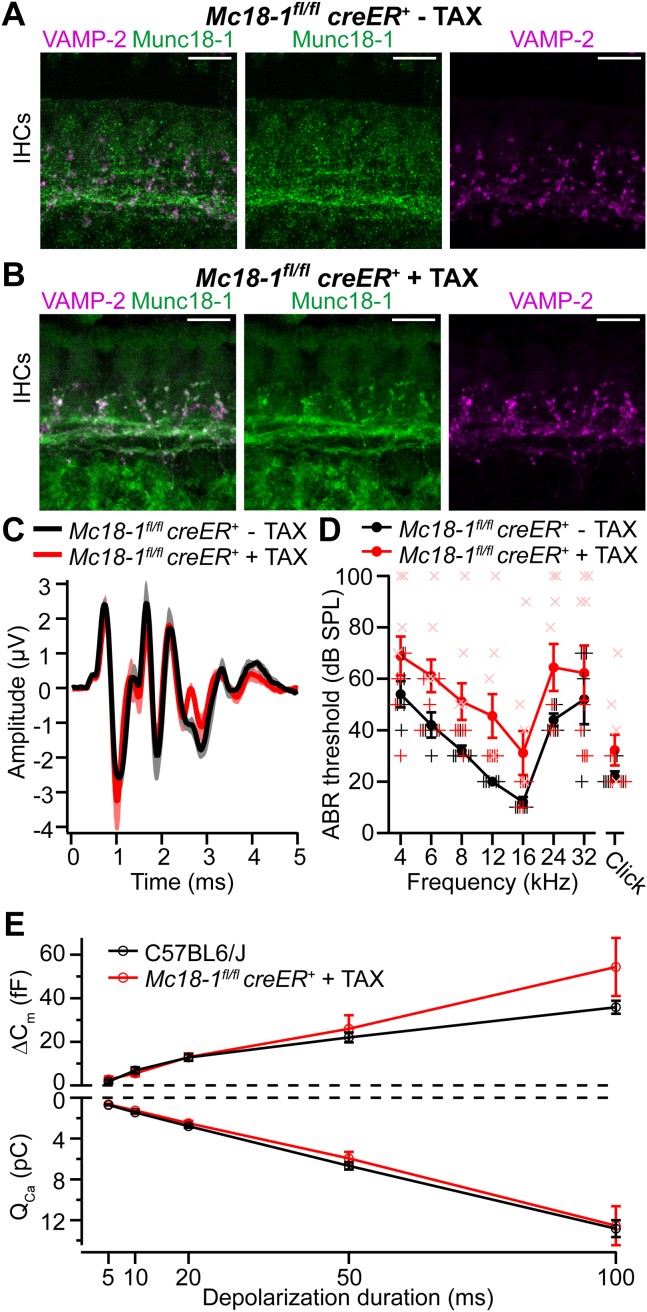
**Conditional knockout of Munc18-1indicates that it is not required for normal function of mouse auditory inner hair cells**. *A*, immunolocalization of Munc18-1 (*green*) in a *Munc18-1*^*fl/fl*^*Math1-creER*^*+*^ conditional knockout mouse that has not been injected with tamoxifen (TAX), resulting in the normal expression of Munc18-1. Co-localization with VAMP-2 (*magenta*) indicates the expression of Munc18-1 in efferent synapses. *B*, injection of tamoxifen leads to activation of creER and conditional knockout of *Munc18-1* in IHCs. The immunolocalization pattern of Munc18-1 (*green*) is not impaired, indicating that the slight labeling of IHCs results from unspecific background staining (scale bars: 10 μm). *C*, auditory brainstem response (ABR) evoked by 80 dB clicks in tamoxifen-injected (red, N = 9 mice) and uninjected (*black*, N = 5 mice) *Munc18-1*^*fl/fl*^*Math1-creER*^*+*^ mice. *D*, ABR audiograms from tamoxifen-injected (*red*, N = 9) and uninjected (*black*, N = 5) *Munc18-1*^*fl/fl*^*Math1-creER*^*+*^ mice. Thresholds measured from individual animals are displayed as crosses and are slightly offset laterally for better visibility. While recordings from one litter of injected animals (*red crosses*, N = 5) indicated normal hearing thresholds as compared to uninjected animals (*black crosses*, N = 5), recordings from a second litter of injected animals (*light red crosses*, N = 4) indicated apparently elevated hearing thresholds. *E*, patch-clamp recording in perforated patch configuration from IHCs of tamoxifen-injected *Munc18-1*^*fl/fl*^*Math1-creER*^*+*^ mice (*red*, n = 8 IHCs from N = 5 mice) indicates normal synaptic transmission from IHCs, since exocytic membrane capacitance increments (ΔC_m_) and calcium currents (I_Ca_) recorded in response to depolarizing pulses of varying duration are comparable to data acquired in B6 wildtype mice (*black*, n = 10 IHCs from N = 5 mice).

Munc18-1 and SNAP-47 were not enriched after hearing onset in VGluT3 immunoisolates when compared to both control IgG immunoisolates and input samples and were, therefore, excluded from our final list of proteins. Nonetheless, given the key role of Munc18-1 at conventional synapses (124), we performed an in-depth analysis of the so far unknown expression and function in the organ of Corti (Fig. 10). *Munc18*-*1* conditional knockout mice (*Mc18-1*^*fl/fl*^) (77) were crossbred with *Math1-CreER* mice and Cre-recombination was induced by tamoxifen injection (*Mc18-1*^*fl/fl*^*/CreER*^+^ + TAX). While Munc18-1 immunofluorescence was obvious in VAMP-2-positive efferent terminals (not affected by Math1-Cre-recombination), it was not clear for IHCs, as the Munc18-1 immunofluorescence with and without TAX-injection appeared similar (Fig. 10, *A* and *B*). Line profile analysis indicated that Munc18-1 immunofluorescence shows a peak outside otoferlin-labeled IHCs, consistent with expression in efferent terminals (supplemental Fig. S8). Recordings of auditory brainstem responses (ABR) did not reveal significant differences between *Mc18-1*^*fl/fl*^*/CreER* mice with and without TAX-injection in ABR sound threshold or ABR wave I amplitude, indicating intact synaptic sound encoding in the absence of Munc18-1 from IHCs (Fig. 10, *C* and *D*). This notion was further supported by perforated-patch-clamp recordings of Ca^2+^ influx-triggered exocytic membrane capacitance increments from IHCs of TAX-injected *Mc18-1*^*fl/fl*^*/CreER* mice that were not different from control IHCs of B6 wildtype mice (Fig. 10*E*). Together this suggests that IHCs do not functionally express Munc18-1, but a (compensatory) expression of Munc18-2 and/or Munc18-3 remains to be tested. Of these, Munc18-2 seems to be expressed in efferent synapses (supplemental Fig. S8). SNAP-47 immunofluorescence (supplemental Fig. S9) was observed in IHCs and in adjacent neurons. Yet, even if expressed in IHCs, efferent SNAP-47 expression could explain the weak enrichment in VGluT3 immunoisolates when compared to the input sample, and hence the exclusion from the final list. The same is true for CaMKIIδ and CaMKIIγ, previously shown to be expressed in IHCs (59).

To further validate our MS data and the reported developmental changes upon hearing onset, in our immunohistochemistry analysis we also investigated the presence of some proteins before hearing onset (at P8-10) and compared it to the immunofluorescence observed after hearing onset (at P15–25) (supplemental Fig. S10). Our immunostainings were positive for syntaxin-7 in mature IHCs only (supplemental Fig. S10*A*), confirming our MS data showing syntaxin-7 enrichment in VGluT3 immunoisolates only after hearing onset at P23. VAMP-7 was enriched at both P8 and P23 in our VGluT3 immunoisolates, and significantly more at P23. We observed immunofluorescence signal for VAMP-7 at both ages but, interestingly, the signal appears more intense and distributed across the cell after hearing onset (supplemental Fig. S10*B*), supporting our MS results. SCAMP1 showed immunofluorescence signal in IHCs at both developmental stages and with no apparent differences (supplemental Fig. S10*A*), supporting our MS data where SCAMP1 was enriched at both P8 and P23. We cannot rule out the possibility that the positive immunolabeling observed for at least some of these proteins could originate from the unspecific binding of the antibodies used. The use of knockout mice, as demonstrated for Munc18-1, in future experiments will help validate the presence of these proteins in IHCs. If validated, super-resolution imaging and immunogold-electron microscopy could help pinpoint the exact location of these newly identified proteins within the IHC, and functional studies could ascertain their relevance in IHC function.

## Discussion

IHCs of the inner ear’s organ of Corti employ an unconventional presynaptic molecular machinery. This is exemplified by the expression of otoferlin, a hair cell-specific multi-C_2_-domain protein, and VGluT3, a vesicular glutamate transporter that is otherwise found in neurons co-releasing glutamate rather than in glutamatergic neurons. Moreover, the SNARE proteins mediating exocytosis at IHC synapses seem to differ from all other neuronal synapses, and they have not been unequivocally identified. It is likely that exocytosis and recycling of synaptic vesicles at these synapses involve additional unique proteins. Previous transcriptomic and proteomic studies (14, 43, 62, 63, 64, 65, 66, 67, 75, 125) provided an overview of the overall transcriptome and proteome of hair cell populations and are breakthroughs at identifying new genes/proteins underlying deafness. Yet, the scarcity of material in the inner ear (*e.g.* approximately only 700 IHCs in the mouse cochlea: (125, 126, 127)) has limited the progress of elucidating the IHC proteome, especially of synaptic vesicles and other IHC-derived membrane fractions. To gain further insight into the composition of the presynaptic machinery in IHCs, we have isolated membrane vesicles containing VGluT3 from the murine organ of Corti and elucidated the proteome of IHCs’ trafficking organelles before and after the onset of hearing (“immature” and “mature”). We established an integrated biochemical and proteomic workflow to immunopurify IHC SVs and other VGluT3-positive vesicular structures from the organs of Corti of mice. We were able to identify ∼900 proteins, both before and after the hearing onset, and observed developmental changes of the trafficking organelle proteome.

In this study, we have established a workflow for the enrichment of membranes involved in the recycling of synaptic vesicles at IHC ribbon synapses. The present study provides the first complete map of the proteome of IHCs under native conditions (*i.e.*, without solubilizing membranes with detergents) at different developmental stages by explicitly targeting the IHC-specific SV-integral protein, VGluT3. Other studies on ribbon- (14) and otoferlin-associated proteomes (43) also included subcellular information. Different from our study, these studies i) did not compare different developmental stages of IHCs and ii) used whole cochleae and detergents for sample preparation. We based our purification approach on established protocols used for the purification of SVs from the brain, where subcellular fractionation is complemented with an affinity-based purification step (68, 69, 70, 71, 72, 73, 74). These approaches have been used successfully to isolate brain-specific SV subsets and endosomes with a high degree of purity. Immunoisolation of trafficking vesicles has two advantages over classical subcellular fractionation protocols: (i) highly enriched fractions can be obtained from heterogeneous starting material in which the targeted organelle population represents only a minor component, allowing for vesicle purification from small amounts of starting material, and (ii) biochemically distinct vesicles can be separated that otherwise have identical biophysical properties (size and density), such as synaptic vesicles carrying different neurotransmitter transporters (96). Combining microdissection of organs of Corti with a subsequent subfractionation workflow and immunoisolation, we were able to substantially reduce the minimum number of animals per biological replicate for obtaining reproducible results (50 mice), and also reduced the complexity of individual subcellular fractions. Despite these advantages, isolating VGluT3-containing vesicles from organs of Corti was highly challenging since the predicted amounts of the targeted vesicles in the starting fraction were likely orders of magnitude lower than in previous immunoisolation experiments, *e.g.*, applied to the brain. Thus it is not surprising that, although a clear enrichment of VGluT3 was achieved, the proteome is likely to be significantly contaminated by proteins/debris derived from other structures. To try to offset this, we resorted to different strategies for validation of our IHC proteome: we (i) measured protein enrichment by MS not only with respect to control beads but also to the starting material (subcellular fraction S2), (ii) compared two different time points, before and after hearing onset, (iii) compared to published expression of IHC and SGNs on the protein or mRNA levels before and after hearing onset, (iv) verified some of the MS hits by immunostaining on whole-mount explants of organs of Corti, and finally, (v) performed functional analysis in conditional (hair cell specific) Munc18-1 knockout mice. While the analysis of protein enrichment relative to both control beads and starting material is advantageous, we acknowledge that this filtering is not done without problems, since we might have missed proteins on one end, and on the other hand we might have assigned contaminant proteins to the enriched fraction. Therefore, we used this filtering as a starting point, and for each hit independent validation is required.

We observed distinct protein composition across subfractions, reminiscent of the trend observed in subcellular fractionation experiments from entire brains. In the fraction used as starting material for the final immunoisolation procedure (subcellular fraction S2), we detected many of the proteins previously reported for IHCs (*e.g.*, VGluT3, otoferlin, AP-2, myosin-VI). The high degree of reproducibility, demonstrated by PCA analysis, confirmed the robustness of our experimental workflow, and it allowed label-free quantitation and comparisons of both known and until now unidentified proteins in the analyzed samples. We confirmed already known IHC SV and trafficking proteins but also identified potential candidates for trafficking and regulated exocytosis in these specialized presynaptic cells. We generated resource databases listing all proteins identified in subcellular fractions before hearing onset (supplemental Table S1), proteins enriched in VGluT3-positive structures before and after hearing onset, quantitative protein rankings, and annotations with subcellular localization and biological function information (supplemental Tables S2–S4).

We found that only ∼27% of the VGluT3-associated proteome is shared between immature and mature IHC. SNARE and SNARE-binding proteins, major players in trafficking, proteins known to be expressed in IHCs (*e.g.*, VGluT3 (52, 53), otoferlin (36), syntaxin-16 (97)) and newly identified proteins (*e.g.*, VAMP-3, VAMP-7) were enriched at both developmental stages but even for these a different degree of enrichment was observed. Other proteins were differentially enriched across developmental stages: VAMP-4, VAMP-5, VAMP-8, and syntaxin-18 before hearing onset; and VAMP-1, syntaxin-6, syntaxin-7, syntaxin-8, syntaxin-12/13, after hearing onset. Additionally, the enrichment only after hearing onset of endocytosis- and endosomal trafficking-related proteins (dynamin-1, dynamin-2, AP-1, AP-2, AP-3) together with the kinase PKCα involved in synaptic transmission and vesicle recycling events in neurons (114, 115, 116, 117), seems to reflect at a molecular level the shift from spontaneous to sound-evoked release activity upon IHC synapse maturation. The classical neuronal SV proteins synaptotagmin-2 and synaptophysin, previously reported to be absent from IHCs (28, 30), were enriched in the proteome analysis at both ages. IHCs showed obvious synaptotagmin-2 immunofluorescence. While this agrees with a previous report (100), we note that two other studies reported a lack of synaptotagmin-2 immunofluorescence from IHCs of mice after hearing onset (30, 32) and that no obvious alteration of IHC exocytosis was found in synaptotagmin-2 knockout mice (30). The synaptophysin signal is localized adjacent to IHCs and, hence, likely is of neuronal origin as previously stated (28).

We cannot entirely rule out the possibility that our proteome analysis was, to some extent, contaminated by adjacent synapses. Nonetheless, the depletion in proteins previously reported to locate to efferent and afferent neurons from our immunoisolates, accompanied by an enrichment in SNAREs and trafficking proteins of mixed SV and endosomal nature points to a distinct molecular signature of IHC exocytosis. Most trafficking proteins identified in mature IHCs were of SV, endosomal, and lysosomal nature. Golgi and ER proteins also make a large part of the identified proteome, but a minority of these were trafficking proteins, and of those a vast majority were proteins involved in trafficking between the two compartments and proteins caught in traffic.

We validated key mass spectrometry hits *via* immunohistochemistry and confocal microscopy. We observed immunofluorescence signal for several SNARE proteins in IHCs which could potentially be involved in trafficking events. Of these, VAMP-7 and syntaxin-12/13 are distributed to compartments close to the basolateral plasma membrane and the synaptic ribbon, and we speculate they could be involved in recycling events. Syntaxin-7 and syntaxin-8 showed a wider distribution across the cell and might be involved in constitutive trafficking. Munc18-1 and SNAP-47, although detected *via* mass spectrometry, were not enriched in our analysis. Munc18-1 does not seem to have a functional role in IHC exocytosis after the onset of hearing. The SNAP-47 expression in IHCs, suggested by immunofluorescence analysis, remains to be validated in future studies employing knockout controls. Likewise, we found immunofluorescence signal for the SV proteins SCAMP1, V-ATPase, and SV2, and of the Golgi and endosomal proteins synaptobrevin homolog YKT6 and Vti1A, the ER membrane receptor for lipid- and sterol-binding protein VAP-A, and the transitional ER ATPase VCP. Yet, these results require further validation by using knockout animals for these proteins, to rule out unspecific antibody binding and to test for a role in IHC presynaptic function.

In IHCs, vesicle recycling is thought to occur at the base of the cell while constitutive trafficking events occur at the apex, but it is still unclear how proteins traffic from the apex to the base of the cells (97, 128). It is possible that the latter occurs from the Golgi apparatus *via* an endosomal network towards the base of the cell, an exciting hypothesis that should be tested in future studies. Efficient vesicle resupply to the ribbon-type active zones is critical for IHC function and occurs primarily *via* recycling from endocytic compartments (60, 61, 97, 128). In fact, IHCs seem to possess a specialized endosomal network located at the base of the cell which was reported to be essential in this process and composed of vesicles and endosomes of different sizes. In this context, otoferlin is seen as a key protein with putative functions in several steps of the SV cycle, from SV exocytosis to SV endocytosis, but also vesicle budding from endosomes and vesicle resupply to the ribbon (33, 39, 42, 43, 44, 60, 113). Our data supports the involvement of other players in one or more of these processes.

Our study represents a first attempt at obtaining a complete proteome of mammalian IHC trafficking organelles. The identification of many proteins in IHCs may pave the way for future physiological studies about their relevance in synaptic transmission. This together with recent advances in the study of molecular mechanisms involved in membrane trafficking events will hopefully contribute to obtaining an average model of IHCs’ trafficking organelles in the future. For example, deep visual proteomics (129) applied to IHCs will help increase the specificity of the analysis to the IHC proteome. Moreover, this approach might, in the future, even allow the elucidation of subcellular proteomic differences. This might help identify differences between VGluT3-containing trafficking organelles of the apical and basal (synaptic) compartment of the IHC. Moreover, it might elucidate differences in the molecular composition of IHC synapses that exhibit major structural and functional heterogeneity (*e.g.* (23, 130, 131, 132)) likely related to the molecular (*e.g.* (54, 133, 134)) and functional (*e.g.* (135, 136)) diversity of their postsynaptic SGNs. Furthermore, acquiring quantitative information about the copy number of proteins or protein complexes is an important task for the future.

## Data Availability

The mass spectrometry proteomics data have been deposited to the ProteomeXchange Consortium (http://proteomecentral.proteomexchange.org) *via* the PRIDE partner repository (137) with the dataset identifier PXD046664.

## Supplemental data

This article contains supplemental data (111).

## Conflict of interest

The authors declare that they have no conflicts of interest with the contents of this article.
