# Supplementary material for: Proteomic Analysis Reveals the Composition of Glutamatergic Organelles of Auditory Inner Hair Cells
Source: Mol Cell Proteomics. 2023 Dec 20;23(2):100704. doi: 10.1016/j.mcpro.2023.100704 (PMC10832297; doi:10.1016/j.mcpro.2023.100704)
Supplement: Supplemental Data [file mmc1.pdf]

*Supplemental Data for*

# Proteomic analysis reveals the composition of glutamatergic organelles of auditory inner hair cells

Andreia P. Cepeda*, Momchil Ninov*, Jakob Neef, Iwan Parfentev, Kathrin Kusch, Ellen Reisinger, Reinhard Jahn**, Tobias Moser**, Henning Urlaub**

*These authors contributed equally to this work.

**Corresponding author. e-mail: henning.urlaub@mpinat.mpg.de (H.U.), tmoser@gwdg.de (T.M.), reinhard.jahn@mpinat.mpg.de (R.J.).

# Table of Contents

**Supplemental Figures ........ S-2**

Supplemental Figure S1. VGluT3 and otoferlin expression in the mature murine organ of Corti at P16. ........ S-2

Supplemental Figure S2. Immunoblot analysis shows the efficiency of the immunoisolation approach. ........ S-3

Supplemental Figure S3. Volcano plots for enrichment analysis of proteins in VGluT3 immunoisolates, before and after hearing onset. ........ S-4

Supplemental Figure S4. Bar plots showing the relative enrichment of proteins in VGluT3 immunoisolates in an age-dependent manner, at P8 and P23. ........ S-5

Supplemental Figure S5. Ranking of significantly enriched proteins in VGluT3 immunoisolates after hearing onset at P23. ........ S-7

Supplemental Figure S6. Evaluation of expression of selected genes of interest in inner hair cells and type 1 spiral ganglion neurons in the developing cochlea. ........ S-8

Supplemental Figure S7. Immunolocalization analysis of Synaptophysin and Synaptotagmin-2 in the organ of Corti before and after hearing onset. ........ S-9

Supplemental Figure S8. Immunolocalization analysis of Munc18-1 to -3 in the adult organ of Corti. ........ S-10

Supplemental Figure S9. Immunolocalization analysis of SNAP-47 in the adult organ of Corti. ........ S-11

Supplemental Figure S10. Immunolocalization analysis of syntaxin-7, VAMP-7, and SCAMP1 in the organ of Corti before and after hearing onset. ........ S-12

## Supplemental Figures

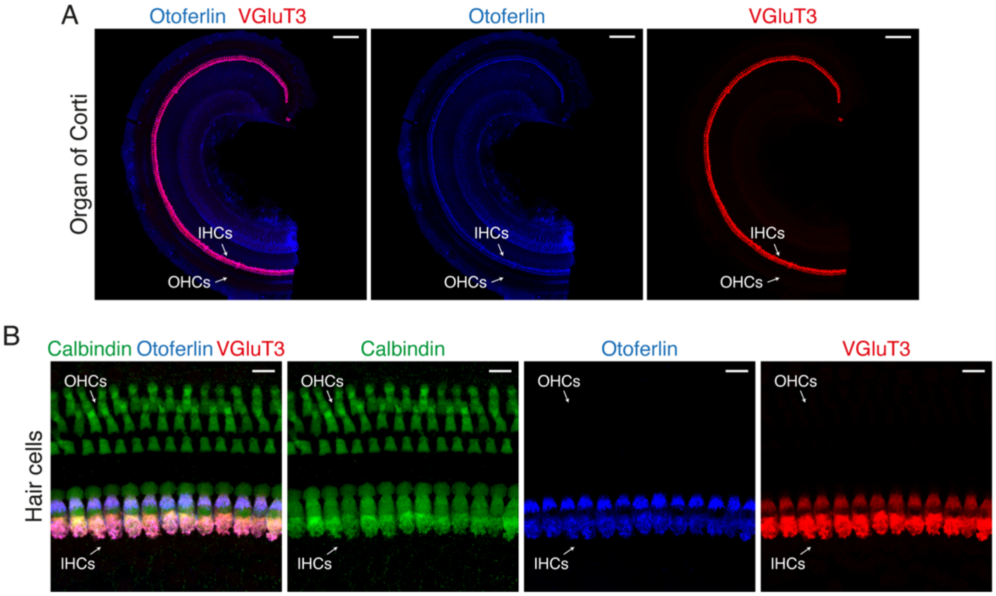


**Supplemental Figure S1. VGluT3 and otoferlin expression in the mature murine organ of Corti at P16.**

**A)** Low magnification views of a representative apical turn of an organ of Corti from a wild-type P16 mouse, immunolabeled for VGluT3 (red) and otoferlin (blue). **B)** High magnification views of IHCs and OHCs from the organ of Corti shown in A. Calbindin was used as hair cell marker. For clarity, individual Calbindin, VGluT3 and otoferlin channels are depicted separately. In (A-B), displayed are maximum intensity projections of confocal optical sections. Scale bars: 100 µm (A), 10 µm (B). IHC, inner hair cell; OHC, outer hair cell.

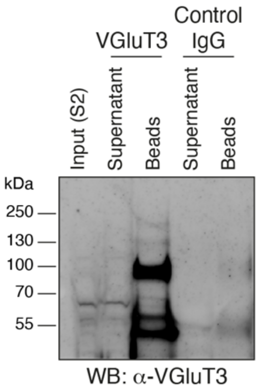


**Supplemental Figure S2. Immunoblot analysis shows the efficiency of the immunoisolation approach.**

Western blot (WB) verification of immunoisolation experiment of VGluT3-positive organelles from P8 organs of Corti. Equal amounts of protein were loaded. Immunoisolation of vesicular structures was performed using magnetic beads coated with a monoclonal mouse VGluT3 antibody. Beads coated with a sheep IgG antibody were used to control for non-specific adsorption. Supernatant S2 (used as input for the immunoisolation experiment) was used as loading control. VGluT3-specific vesicular structures were largely enriched in VGluT3 immunoisolates (VGluT3 beads) and depleted in control IgG experiments (Control IgG beads). Input (S2), S2 fraction; Beads, immunoisolated sample; Supernatant, supernatant after immunoisolation (i.e., unbound proteins).

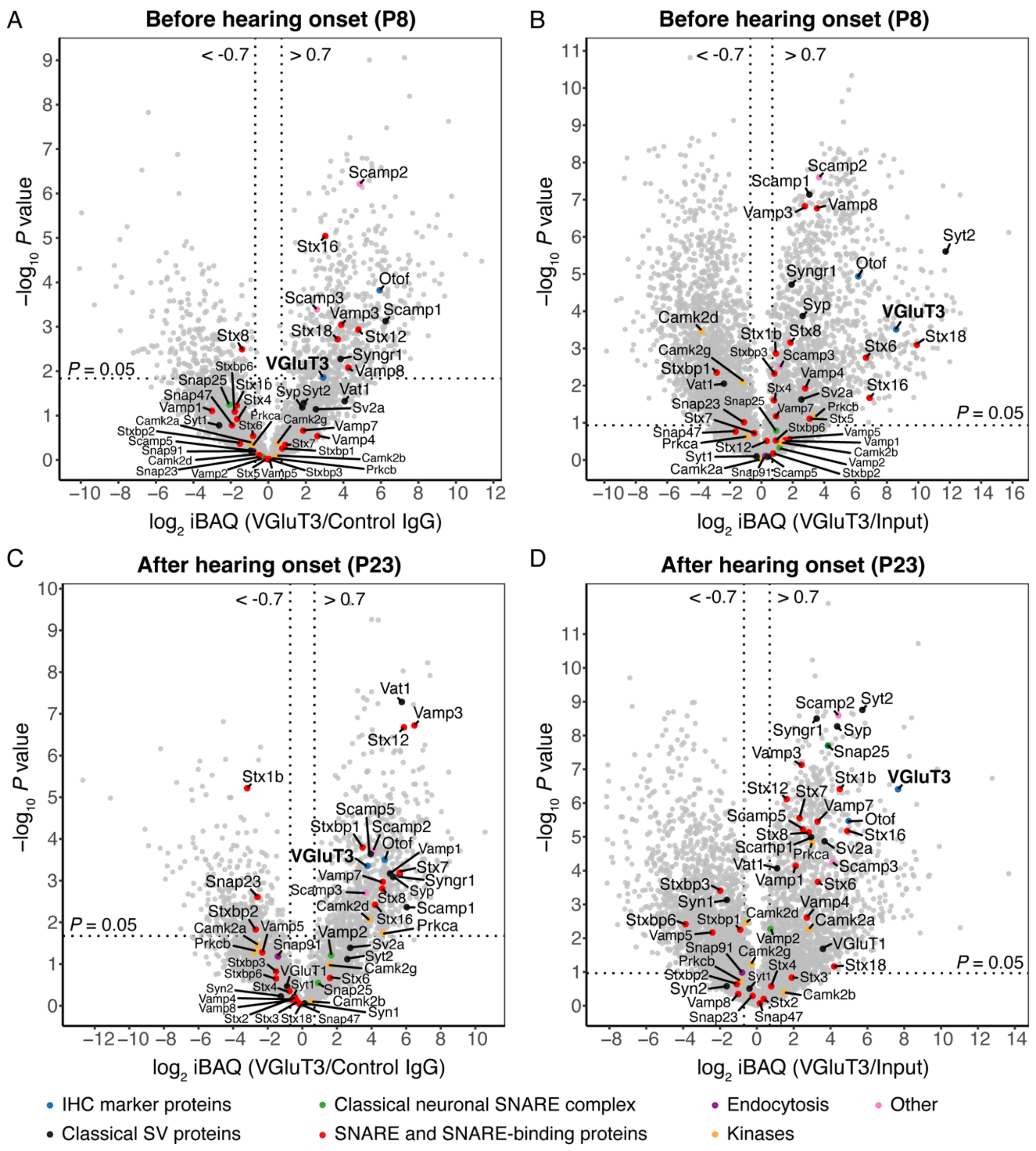


**Supplemental Figure S3. Volcano plots for enrichment analysis of proteins in VGluT3 immunoisolates, before and after hearing onset.**

Volcano plots illustrating differentially enriched proteins in VGluT3 immunoisolates vs. control IgG immunoisolates **(A, C)** or input S2 **(B, D)**, before **(A, B)** and after **(C, D)** hearing onset (at P8 and P23, respectively). The $-\log_{10}$ adjusted *P* value was plotted against the $\log_2$ iBAQ fold change of VGluT3 over control IgG or Input, with a significant *t* test FDR threshold of 5% and $S_0$ = 0. A threshold of $\log_2$ iBAQ fold difference of < -0.7 and > 0.7 was set to consider only proteins with at least 1.5-fold enrichment. Source data are available online for this figure (**supplemental Table S2**). See also **Figure 4** for correlation plots $\log_2$ iBAQ fold change (VGluT3/Control IgG) *vs.* $\log_2$ iBAQ fold change (VGluT3/Input). IHC, inner hair cell; SV, synaptic vesicle.

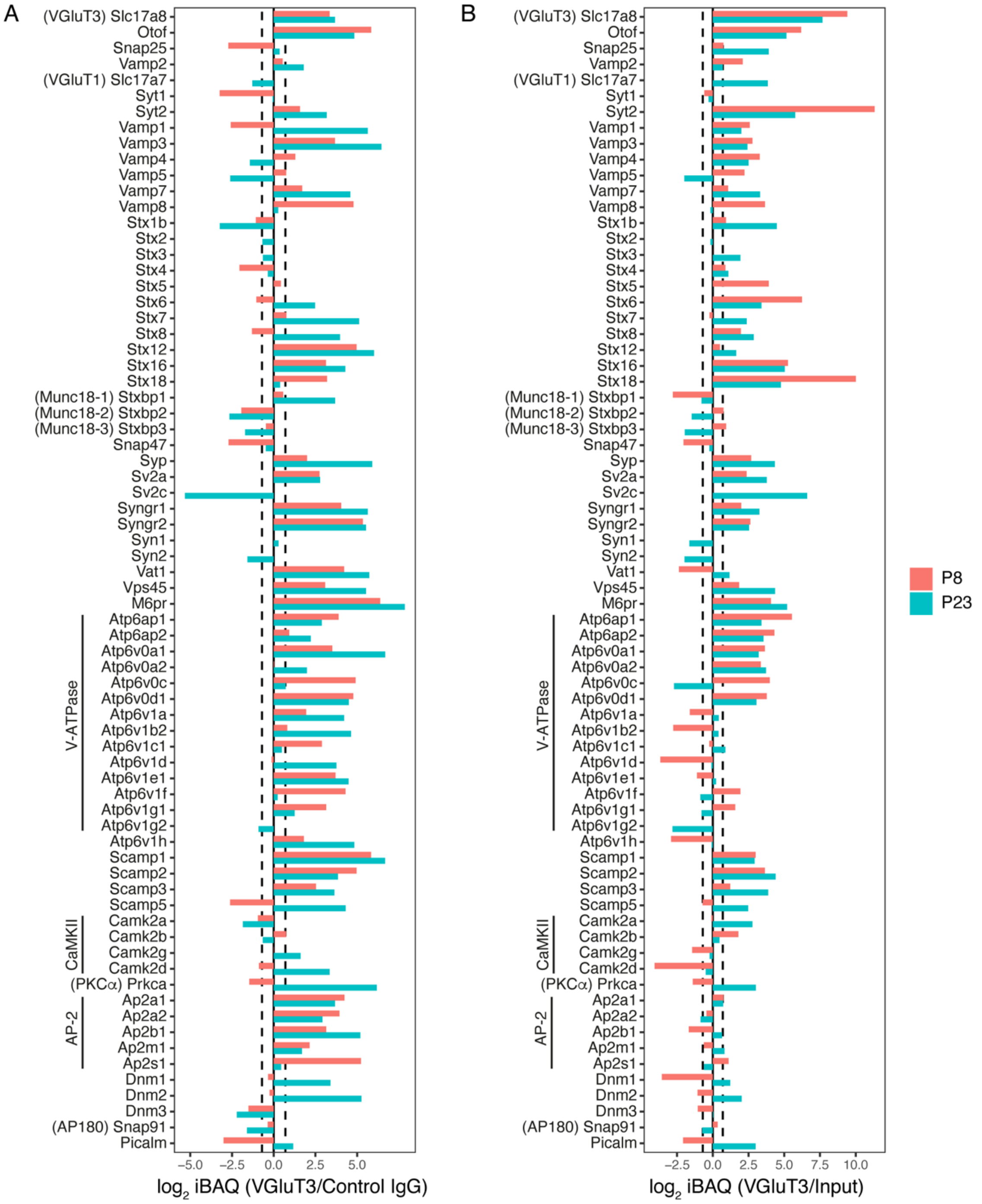


**Supplemental Figure S4. Bar plots showing the relative enrichment of proteins in VGluT3 immunoisolates in an age-dependent manner, at P8 and P23.**

Displayed are IHC marker proteins (e.g., VGluT3 and otoferlin), classical SV proteins, SNAREs, SNARE-binding proteins, kinases, proteins involved in endocytosis, and other trafficking proteins. Protein enrichment in VgluT3 immunoisolates over Control IgG **(A)** and in VgluT3 immunoisolates over Input S2 **(B)**, at P8 (before hearing onset; orange) and P23 (after hearing onset; green). Gene names are displayed. In parentheses are displayed the common protein names for some of the genes. Absent bars indicate that the protein was not identified in that sample. Vertical dashed lines

indicate the threshold of $log_2$ iBAQ fold difference of < -0.7 and > 0.7. See also **Figure 4** for correlation plots $log_2$ iBAQ fold change (VGluT3/Control IgG) *vs.* $log_2$ iBAQ fold change (VGluT3/Input). IHC, inner hair cell; SV, synaptic vesicle.

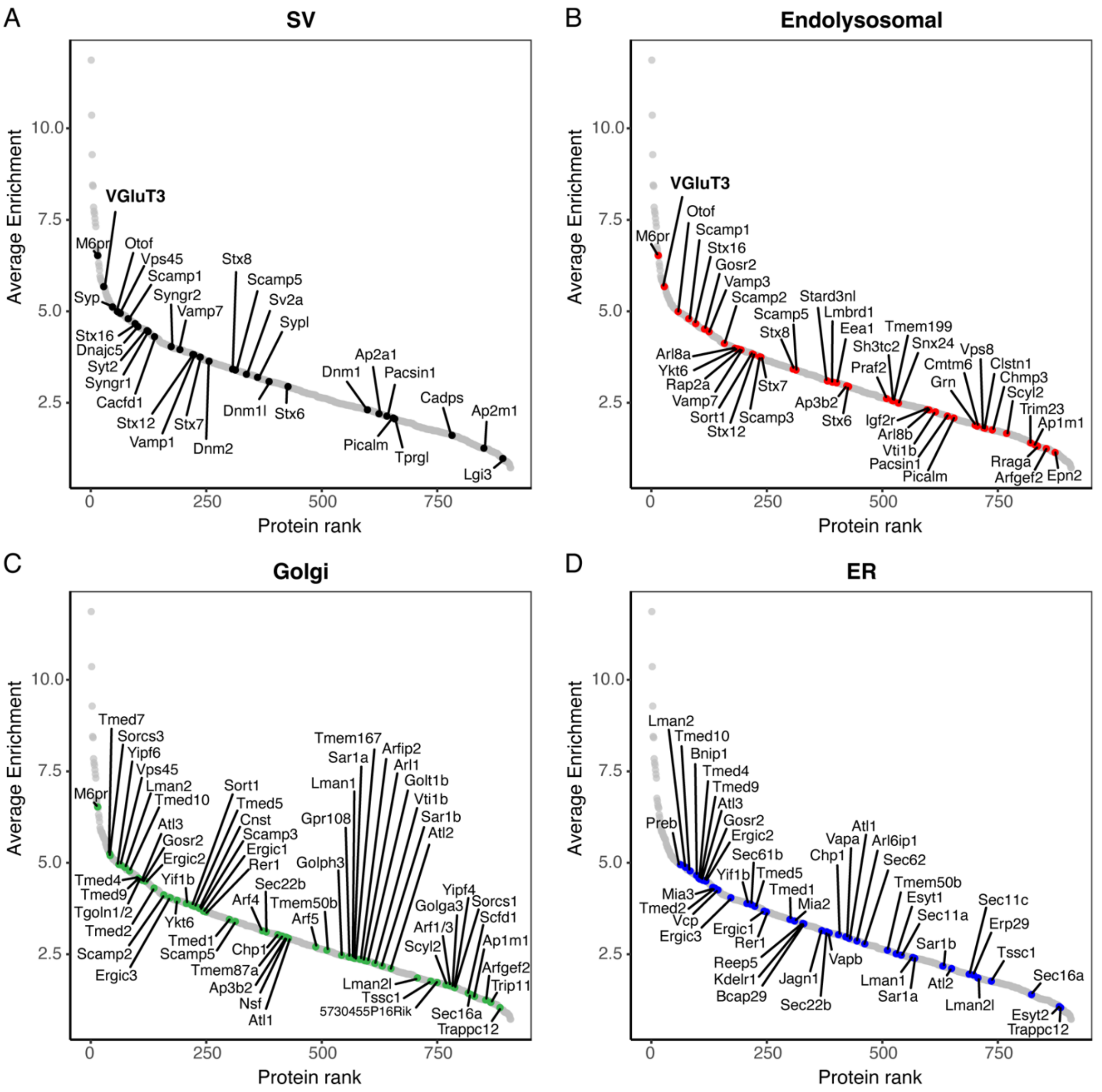


**Supplemental Figure S5. Ranking of significantly enriched proteins in VGluT3 immunoisolates after hearing onset at P23.**

Ranking of proteins in VGluT3 immunoisolates according to their relative enrichment over control IgG immunoisolates and input. Average Enrichment corresponds to the average of $log_2$ iBAQ (VGluT3/Control IgG) and $log_2$ iBAQ (VGluT3/Control IgG). Displayed are proteins involved in trafficking events in different trafficking organelles: SV **(A)**, endolysosomal **(B)**, Golgi **(C)**, and ER **(D)** proteins, including SNAREs and resident proteins. Gene names are displayed. SV, synaptic vesicle; ER, endoplasmic reticulum; PM, plasma membrane.

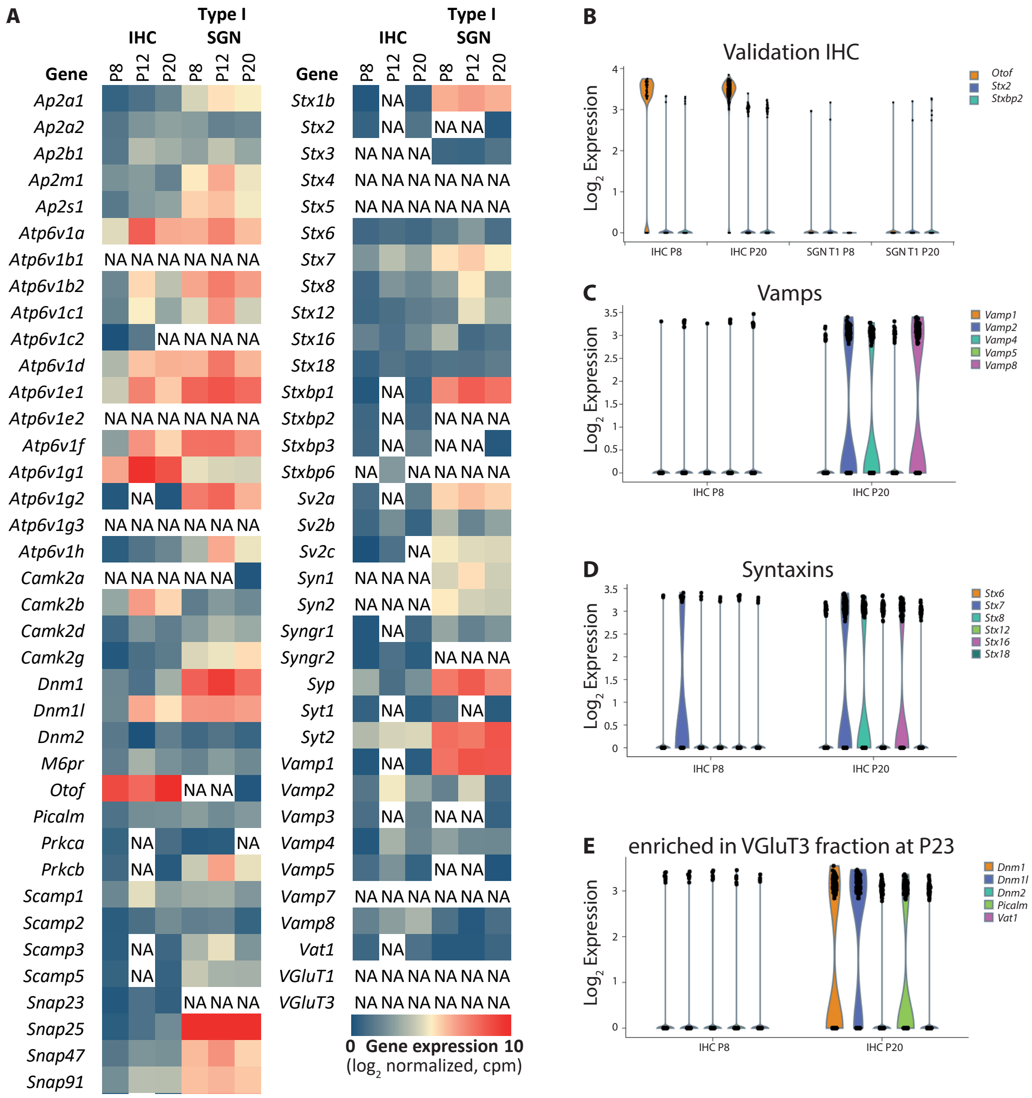


**Supplemental Figure S6. Evaluation of expression of selected genes of interest in inner hair cells and type 1 spiral ganglion neurons in the developing cochlea.**

**A)** Gene expression data inner hair cells (IHCs) and type 1 spiral ganglion neurons (Type I SGN) was extracted from 10x single cell RNA sequencing dataset published by Jean *et al.* 2023 and deposited at umgear.org. Color code representing mean gene expression per cell type given as normalized $log_2$ data in cpm (blue: low expression to red: high expression). **B)** Violin plot depicting robust expression of syntaxin-2 (*Stx2*) and Munc18-2 (*Stxbp2*) in IHCs at P20, but below detection threshold for most IHCs at P8 and SGN at both ages. Otoferlin (*Otof*) as IHC marker protein is given as reference. **C)** Violin plot of *Vamp 1, 2, 4, 5* and *8* expression. Expression of all VAMPs analyzed was increased in IHCs at P20 compared to P8. **D)** Violin plot of syntaxin 6, 7, 8, 12, 16 and 18 expression. While *Stx7* was robustly expressed at P8, expression of the other syntaxins analyzed was sparse at that age, and expression of all syntaxin genes was increased at P20. **E)** Violin plot for genes of proteins enriched in VGluT3 immunoisolates at P23 only (*Dnm1, Dnm1l, Dnm2, Picalm and Vat1*). Compared to IHCs at P8, expression of all genes was clearly induced at P20.

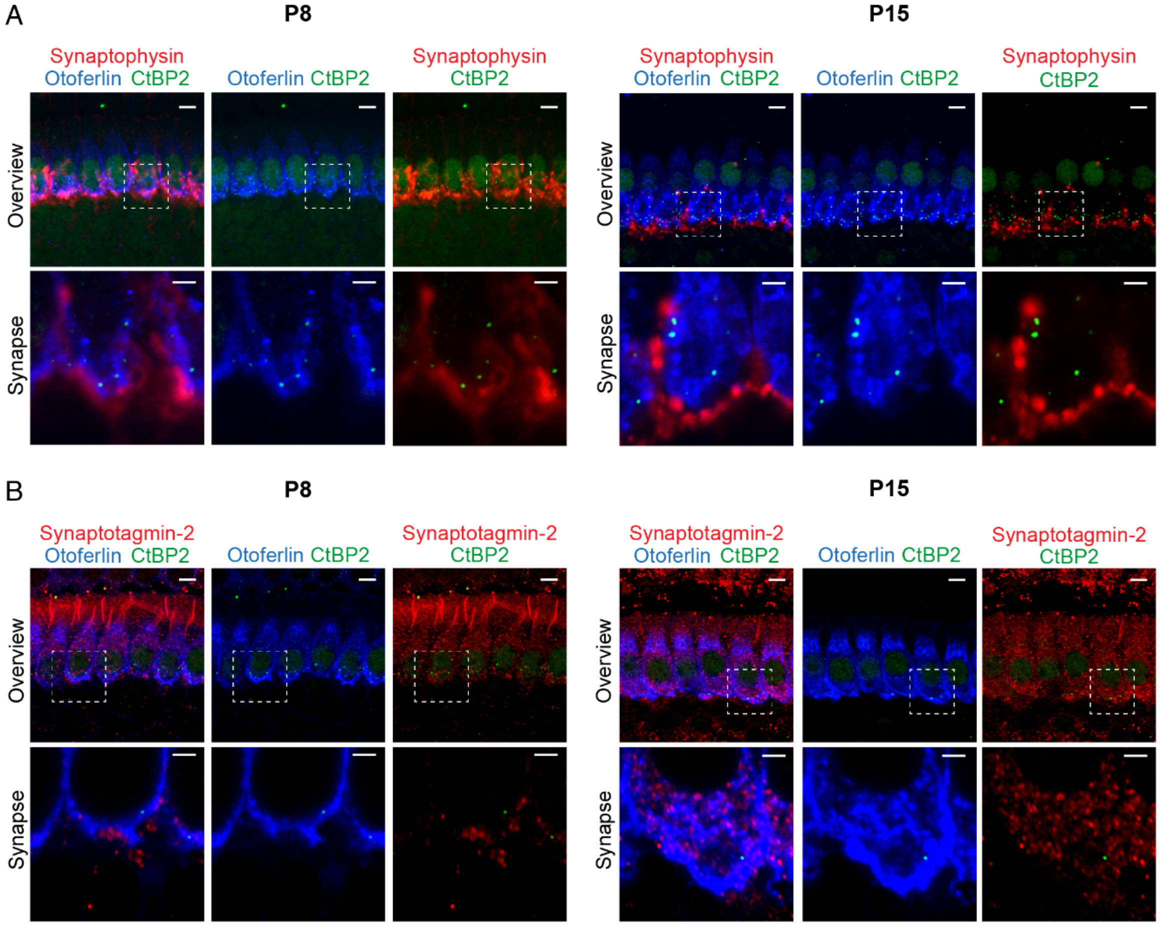


**Supplemental Figure S7. Immunolocalization analysis of Synaptophysin and Synaptotagmin-2 in the organ of Corti before and after hearing onset.**

Expression of synaptophysin **(A)** and synaptotagmin-2 **(B)** in the organ of Corti before (at P8) and after (at P15) hearing onset. Both proteins were enriched in VGluT3 immunoisolates in MS experiments at both ages. Images correspond to high magnification views of representative IHCs immunolabeled with antibodies against the candidate proteins (*red*), the ribbon marker CtBP2/RIBEYE (*green*), and the IHC marker otoferlin (*blue*). Upper panels show overviews of representative IHCs, displaying maximum intensity projections of 5–10 confocal optical sections through the longitudinal axis of the IHCs (scale bars: 5 µm). Bottom panels show a zoom into the synaptic area, displaying single confocal optical sections through the longitudinal axis of a single IHCs at the basal region (scale bars: 2 µm). IHC, inner hair cell; MS, mass spectrometry.

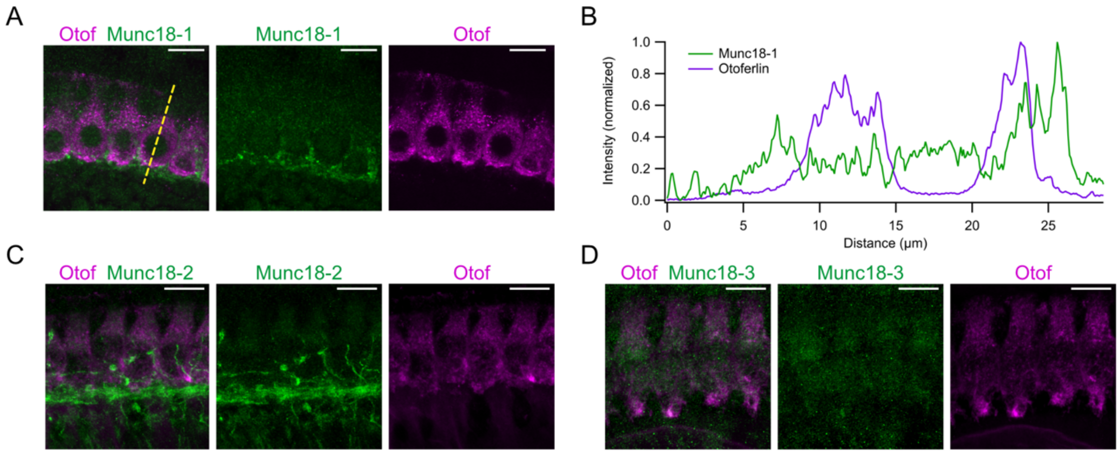


**Supplemental Figure S8. Immunolocalization analysis of Munc18-1 to -3 in the adult organ of Corti.**

Expression of Munc18-1, -2, and -3 as well as of otoferlin in the mature murine organ of Corti. **A)** Maximum intensity projection of IHCs from the apical turn of the organ of Corti of a B6 mouse, immunolabeled for Munc18-1 (*green*) and otoferlin (*magenta*). **B)** Normalized fluorescence intensity along the line profile indicated in A. **C, D)** Maximum intensity projection of IHCs from the apical turn of the organ of Corti of B6 mice, immunolabeled for Munc18-2 (C, *green*) and Munc18-3 (D, *green*) as well as otoferlin (*magenta*) (scale bars: 10 µm). For clarity, individual Munc18 and otoferlin channels are depicted separately. Otof, otoferlin; IHC, inner hair cell.

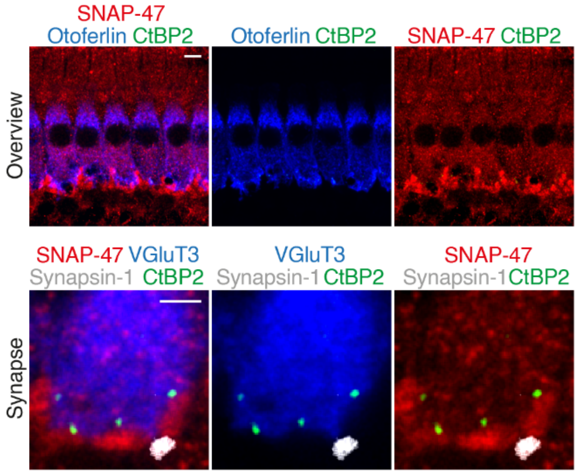


**Supplemental Figure S9. Immunolocalization analysis of SNAP-47 in the adult organ of Corti.**

SNAP-47 was identified but not enriched in VGluT3 immunoisolates, however its expression was confirmed in IHCs and SGN terminals. Images correspond to high magnification views of representative P15–25 IHCs immunolabeled with an antibody against SNAP-47 (*red*), the ribbon marker CtBP2/RIBEYE (*green*), and the IHC markers VGluT3 or otoferlin (*blue*). Upper panels show overviews of representative IHCs, displaying maximum intensity projections of 5–10 confocal optical sections through the longitudinal axis of the IHCs (scale bars: 5 µm). Bottom panels show a zoom into the synaptic area, displaying single confocal optical sections through the longitudinal axis of a single IHCs at the basal region (scale bars: 2 µm). IHC, inner hair cell; MS, mass spectrometry; SGN, spiral ganglion neuron.

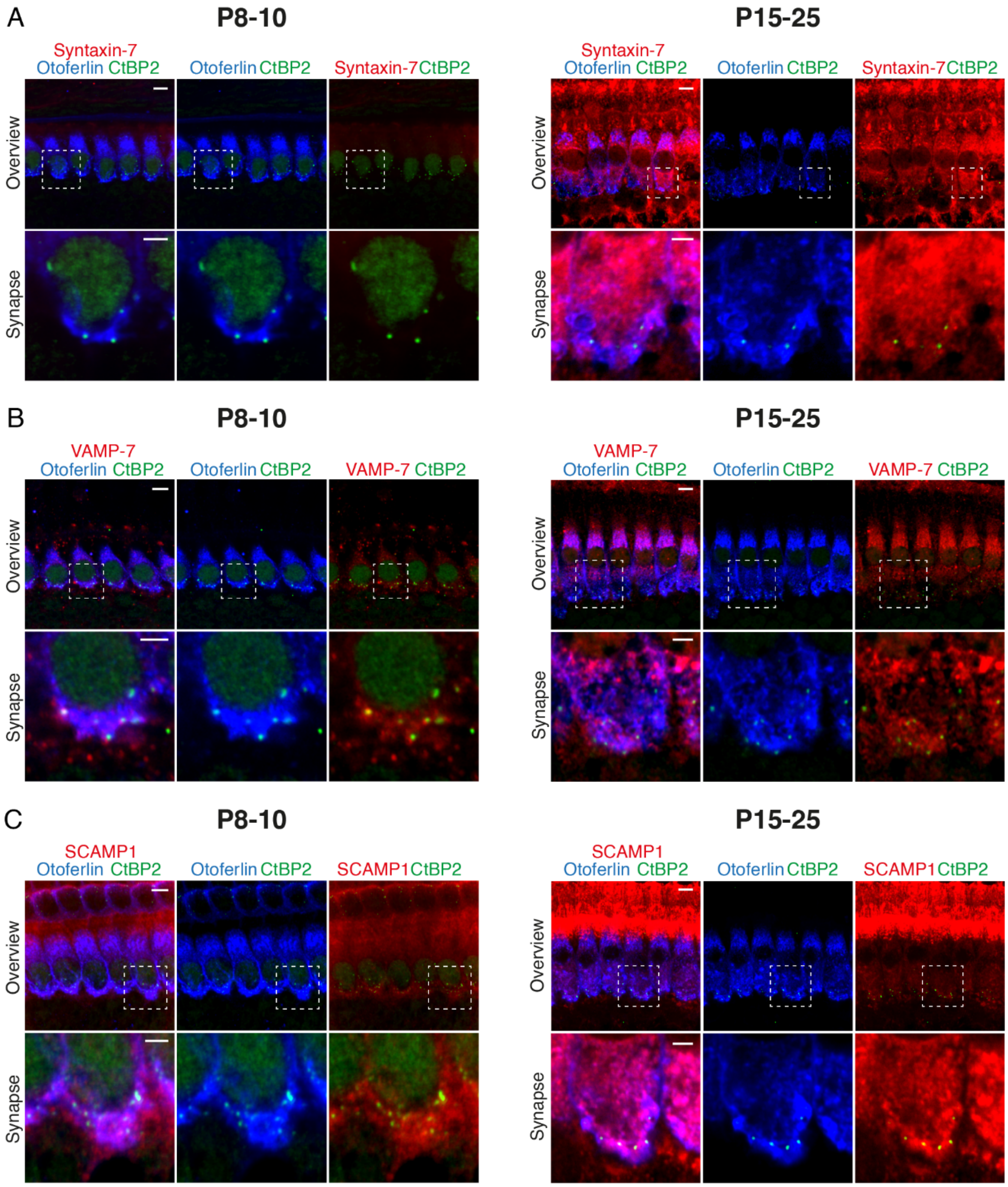


**Supplemental Figure S10. Immunolocalization analysis of syntaxin-7, VAMP-7, and SCAMP1 in the organ of Corti before and after hearing onset.**

Expression of syntaxin-7 **(A)**, VAMP-7 **(B)**, and SCAMP1 **(C)** in the organ of Corti before (at P8-10) and after (at P15-25) hearing onset. Syntaxin-7 was enriched in VGluT3 immunoisolates in MS experiments after hearing onset only (at P23), while VAMP-7 and SCAMP1 were enriched both before and after hearing onset. Images correspond to high magnification views of representative IHCs immunolabeled with antibodies against the candidate proteins (*red*), the ribbon marker CtBP2/RIBEYE (*green*), and the IHC marker otoferlin (*blue*). Upper panels show overviews of representative IHCs, displaying maximum intensity projections of 5–10 confocal optical sections through the longitudinal axis of the IHCs (scale bars: 5 μm). Bottom panels show a zoom into the

synaptic area, displaying single confocal optical sections through the longitudinal axis of a single IHCs at the basal region (scale bars: 2 μm). IHC, inner hair cell; MS, mass spectrometry.
